# Supplementary material for: Design, Synthesis and Evaluation of Novel 1,4-Disubstituted Piperazine-2,5-dione Derivatives as Antioxidants against H2O2-Induced Oxidative Injury via the IL-6/Nrf2 Loop Pathway
Source: Antioxidants (Basel). 2022 Oct 12;11(10):2014. doi: 10.3390/antiox11102014 (PMC9598289; doi:10.3390/antiox11102014)

Supporting Information for

Design, Synthesis and Evaluation of Novel 1,4-Disubstituted Piperazine-2,5-dione Derivatives as Antioxidants against H_2O_2-Induced Oxidative Injury via the IL-6/Nrf2 Loop Pathway

Liang Xiong [1,2,†], Hongshan Wu [1,2,†], Ting Zhong [1,2], Fang Luo [1,2], Qing Li [1,2], Mei Li [1,2] and Yanhua Fan [1,2,*]

[1] State Key Laboratory for Functions and Applications of Medicinal Plants, Guizhou Medical University, Guiyang 550014, China

[2] The Key Laboratory of Chemistry for Natural Products of Guizhou Province and Chinese Academy of Sciences, Guiyang 550014, China

[*] Correspondence: yhfan@gzcnp.cn

† These authors contributed equally to this work.

Table S1

The impact of synthetic compounds on SH-SY5Y cytotoxicity

Compd.	Viabilities of SH-SY5Y Cells		
	80(μM)	20(μM)	5(μM)
9a	109.1±5.61	104.6±4.71	93.05±8.58
9b	84.20±5.85	109.7±2.65	113.2±3.52
9c	111.6±8.11	130.7±19.42	85.87±10.59
9d	109.8±6.67	115.7±3.96	106.8±5.66
9e	120.4±4.13	108.3±2.33	114.5±2.77
9f	91.23±3.47	120.3±5.16	92.88±3.81
9g	101.1±0.94	97.02±0.68	107.6±2.64
9h	95.36±0.64	106.5±1.23	115.2±1.18
9i	103.7±0.93	81.51±5.06	91.87±2.93
9j	100.3±1.47	107.9±1.08	109.7±1.05
9k	105.6±1.01	99.27±1.33	117.9±1.74
9l	112.5±1.52	114.3±1.03	93.57±6.01
9m	98.77±2.21	101.4±2.64	117.3±7.96
9n	113.1±6.19	107.7±8.49	98.65±1.11
9o	107.7±5.38	113.4±6.76	113.5±6.58
9p	117.9±4.23	112.6±3.85	114.3±5.58
9q	103.1±1.56	107.6±1.01	106.5±2.95
9r	114.1±7.61	119.3±8.86	95.25±4.85
9s	101.8±0.69	101.1±0.74	109.3±3.66

^{1}H, ^{13}C, ^{19}F NMR spectra and HR-MS (ESI) of compounds

*1-(3,5-dimethoxyphenyl)-4-(4-methoxybenzoyl)piperazine-2,5-dione (**9a**)*

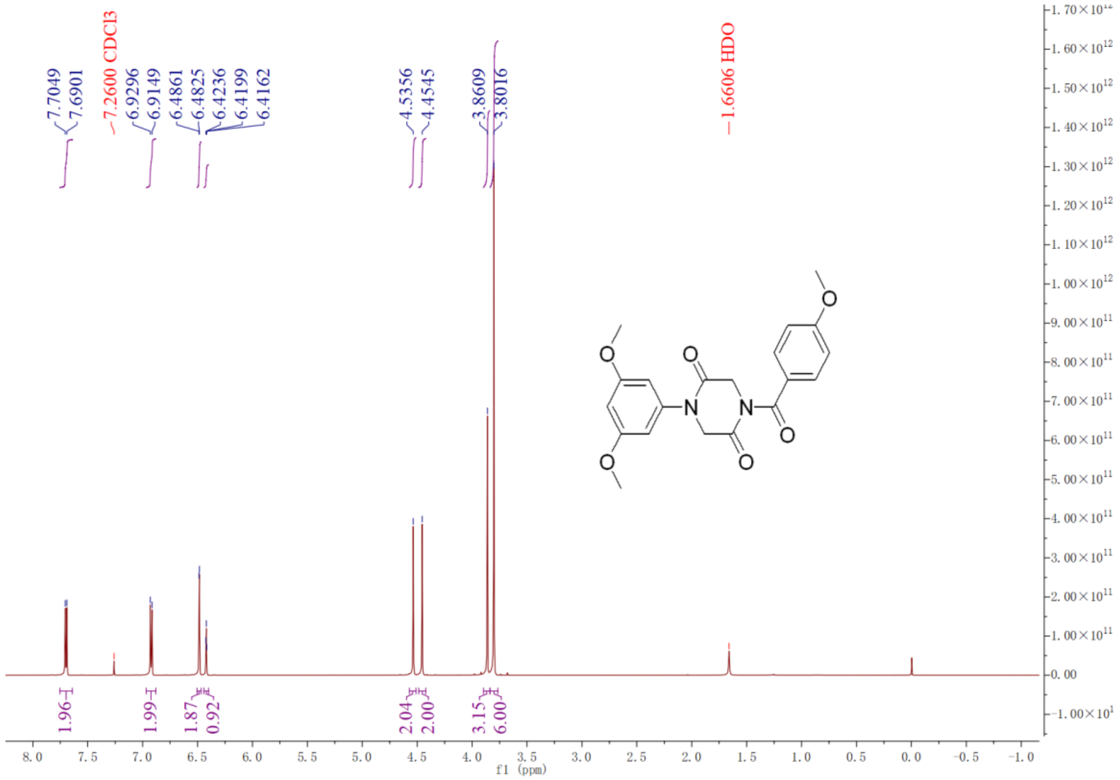
7.7049
7.6901
7.2600 CDCl3
6.9296
6.9149
6.4861
6.4825
6.4236
6.4199
6.4162
4.5356
4.4545
3.8609
3.8016
1.6606 HDO
1.96
1.99
1.87
0.92
2.04
2.00
3.15
6.00
f1 (ppm)

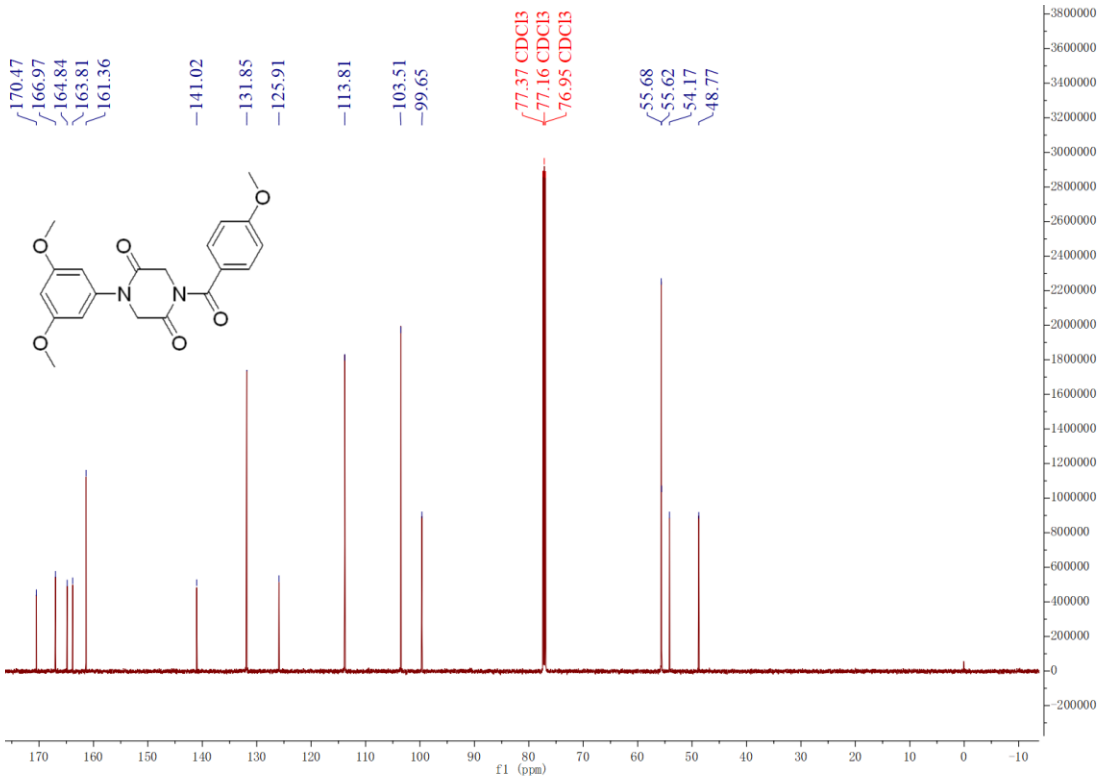
170.47
166.97
164.84
163.81
161.36
141.02
131.85
125.91
113.81
103.51
99.65
77.37 CDCl3
77.16 CDCl3
76.95 CDCl3
55.68
55.62
54.17
48.77
f1 (ppm)

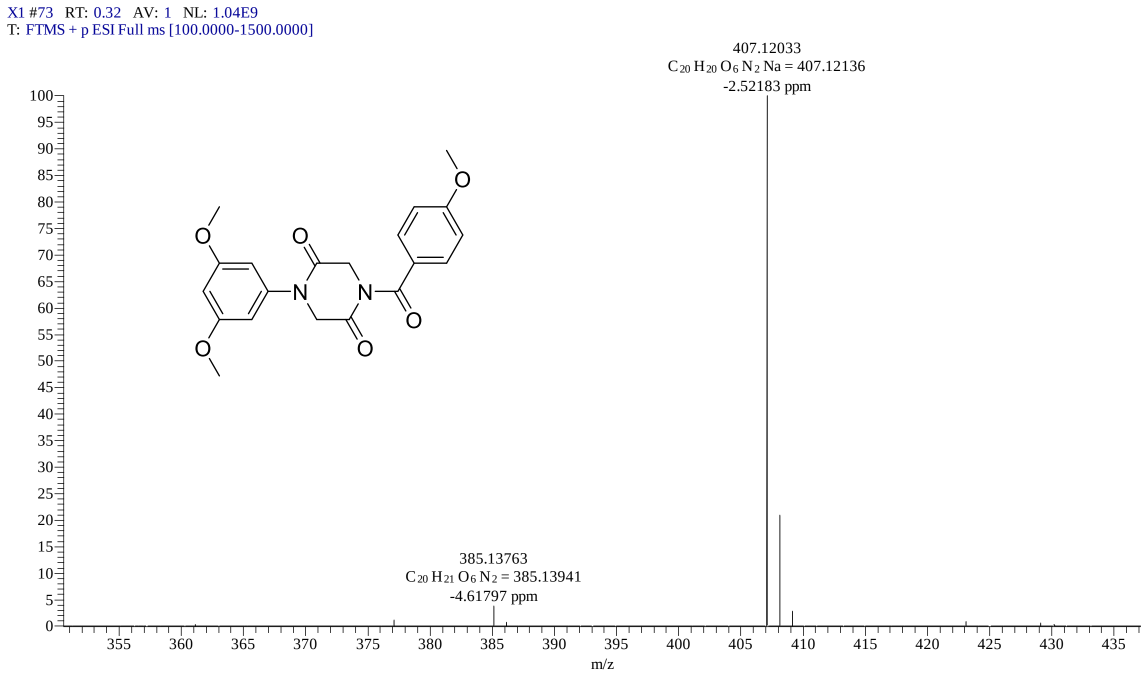

*1-(3,5-dimethoxyphenyl)-4-(4-methylbenzoyl)piperazine-2,5-dione (**9b**)*

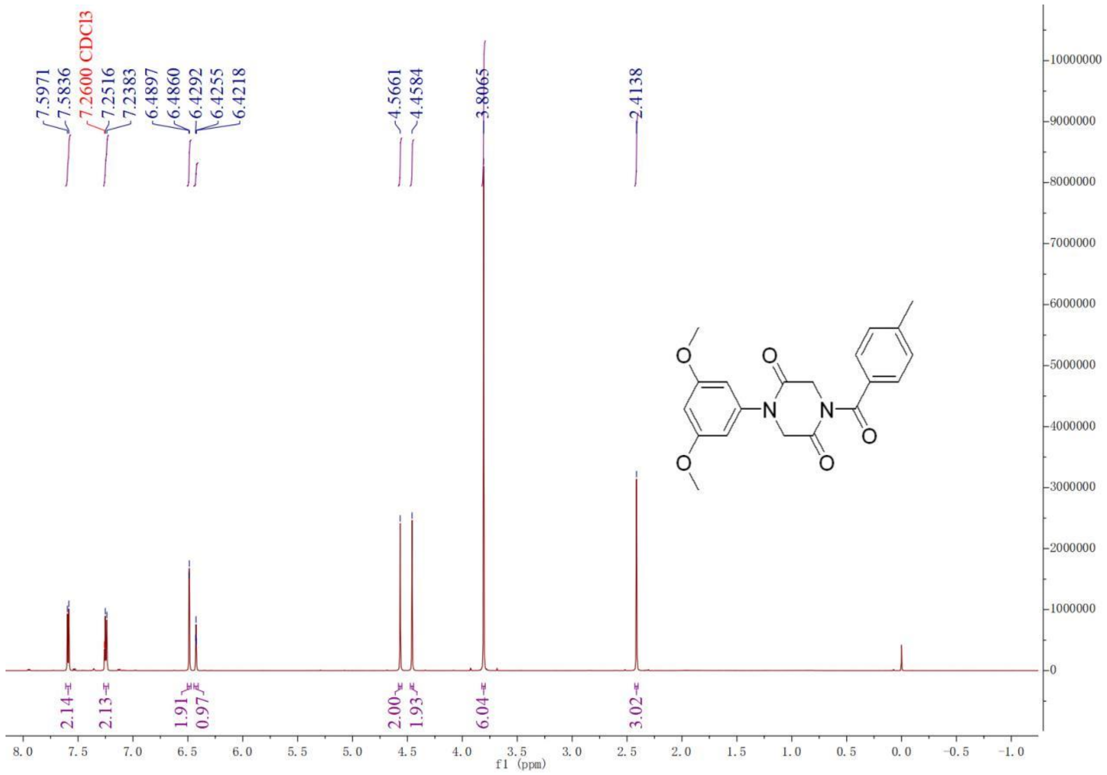

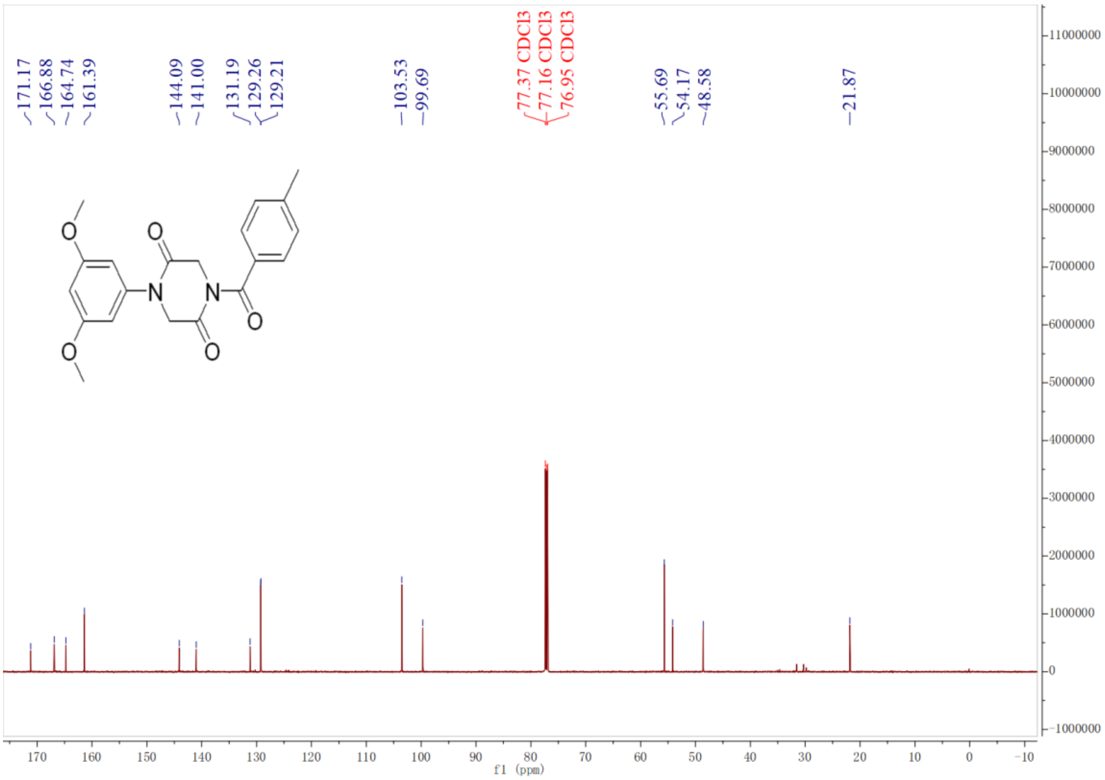

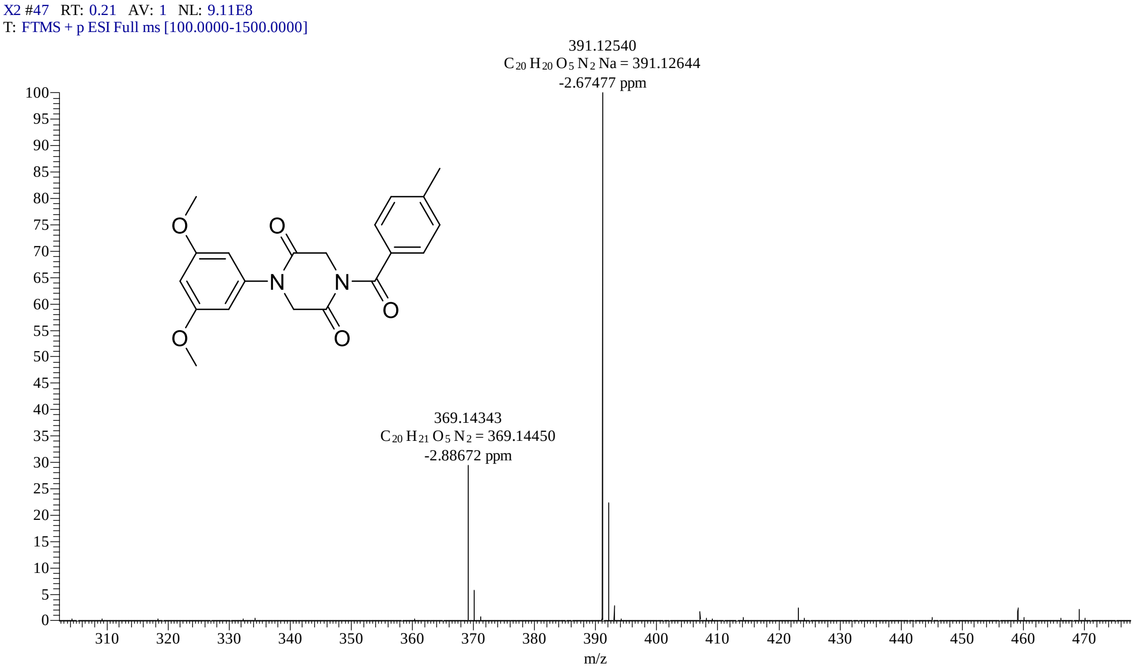

*1-(4-methylbenzoyl)-4-(3,4-methylenedioxyphenyl)piperazine-2,5-dione (**9c**)*

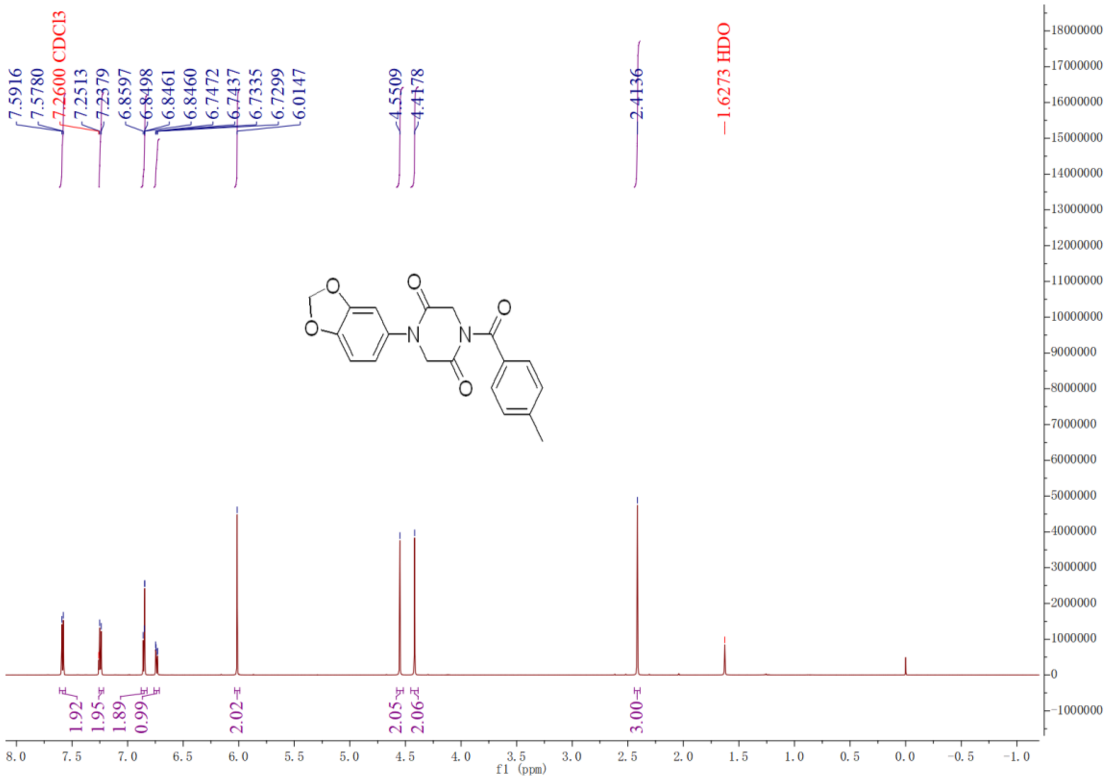

7.5916
7.5780
7.2600 CDCl3
7.2513
7.2379
6.8597
6.8498
6.8461
6.8460
6.7472
6.7437
6.7335
6.7299
6.0147
4.5509
4.4178
2.4136
1.6273 HDO
1.92
1.95
1.89
0.99
2.02
2.05
2.06
3.00
f1 (ppm)

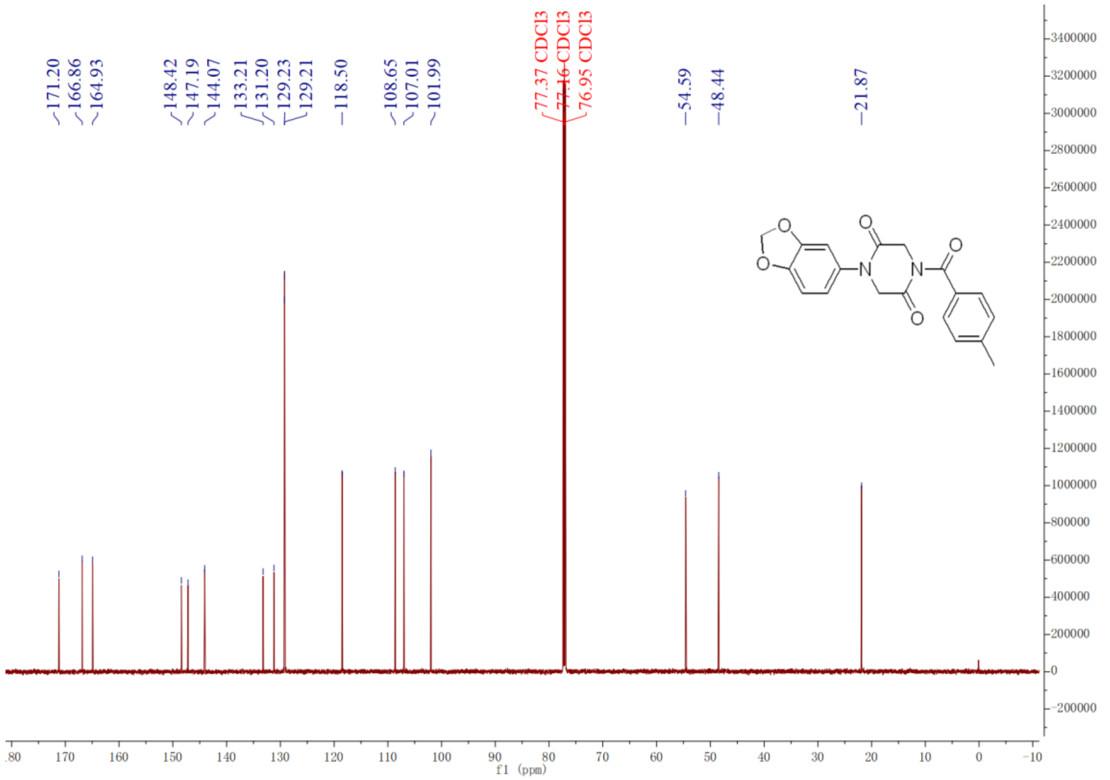

171.20
166.86
164.93
148.42
147.19
144.07
133.21
131.20
129.23
129.21
118.50
108.65
107.01
101.99
77.37 CDCl3
77.16 CDCl3
76.95 CDCl3
54.59
48.44
21.87
f1 (ppm)

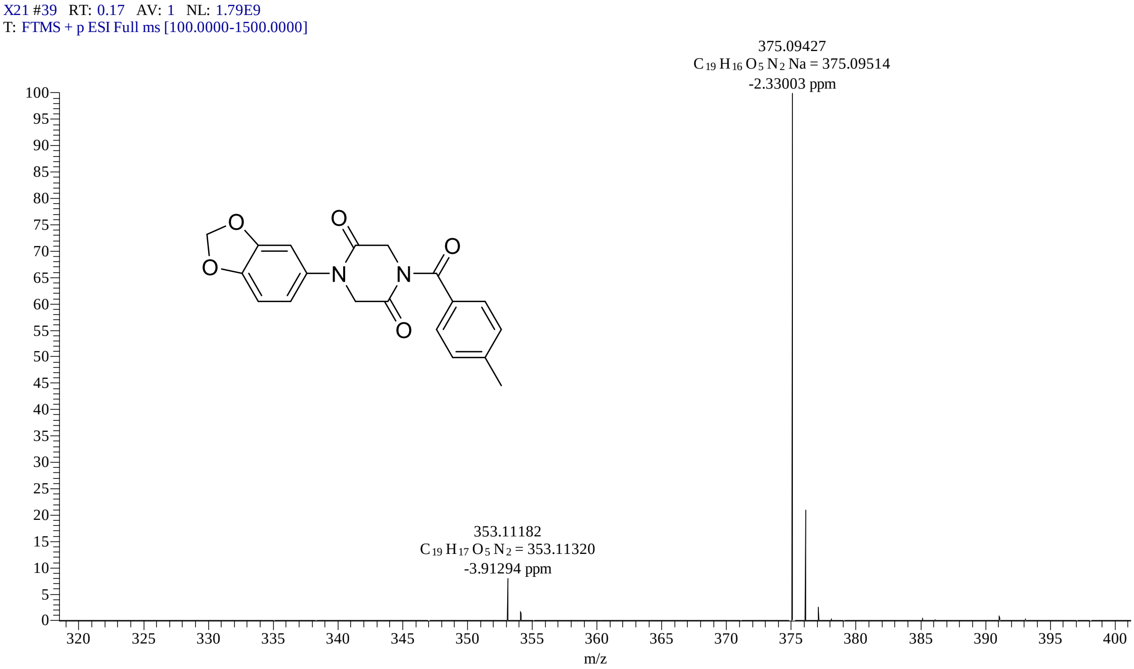

*1-(4-methylbenzoyl)-4-phenylpiperazine-2,5-dione (**9d**)*

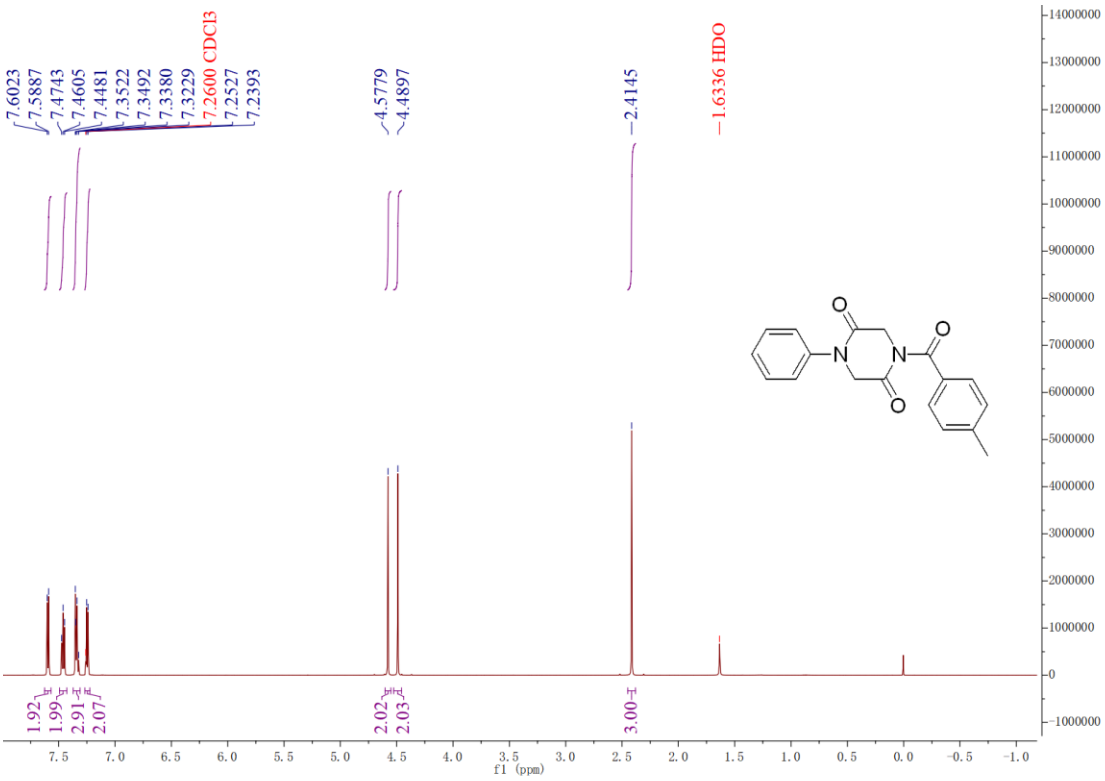

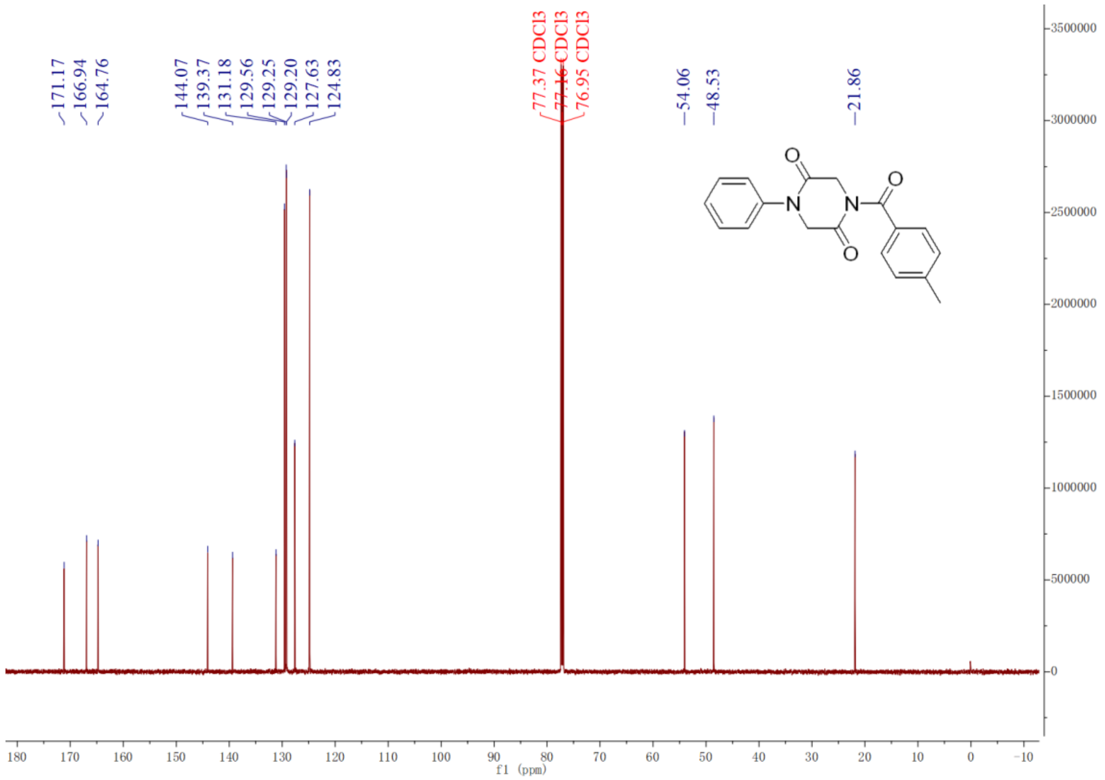

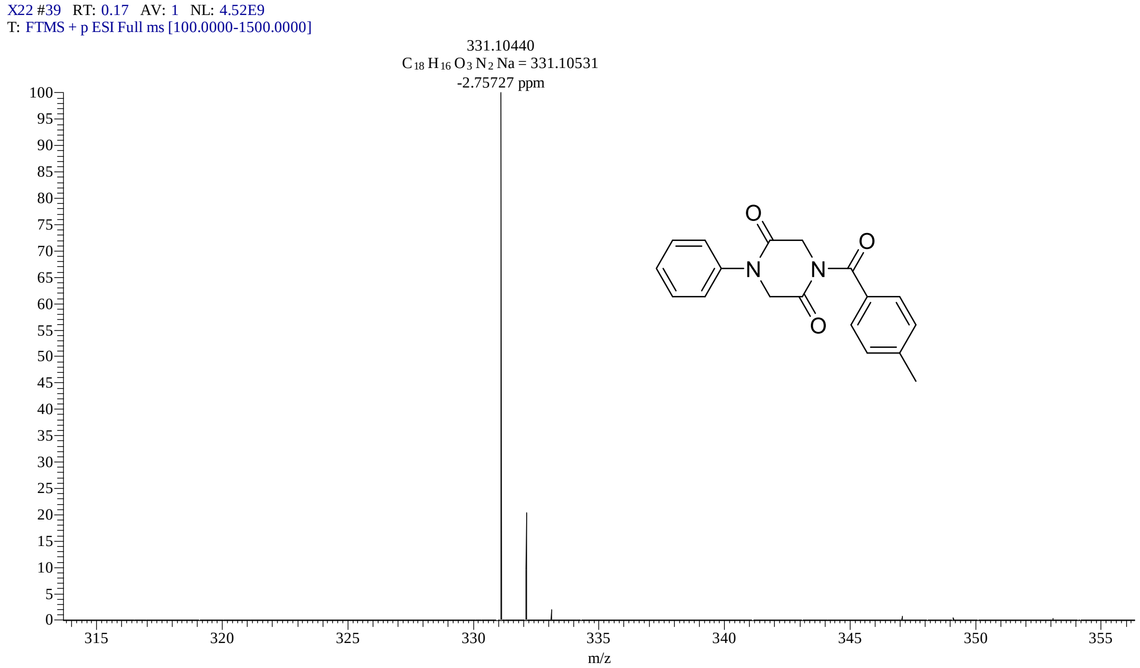

*1-(3-methoxyphenyl)-4-(4-methylbenzoyl)piperazine-2,5-dione (**9e**)*

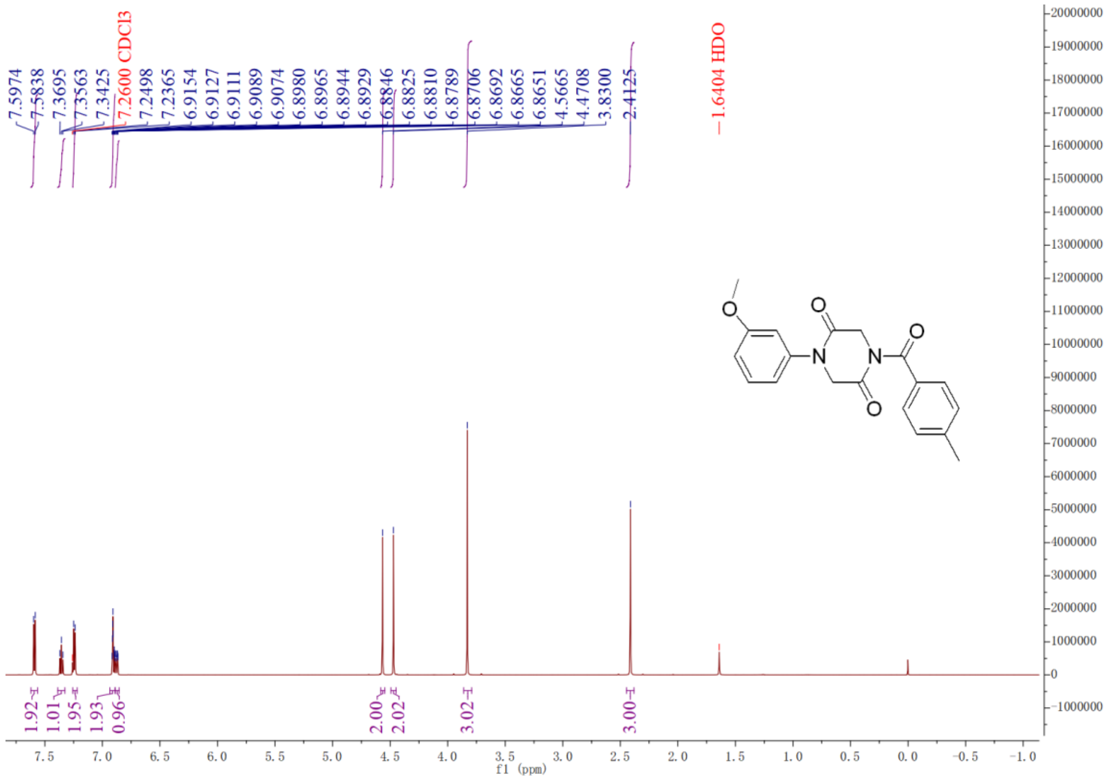
7.5974
7.5838
7.3695
7.3563
7.3425
7.2600 CDCl3
7.2498
7.2365
6.9154
6.9127
6.9111
6.9089
6.9074
6.8980
6.8965
6.8944
6.8929
6.8846
6.8825
6.8810
6.8789
6.8706
6.8692
6.8665
6.8651
4.5665
4.4708
3.8300
2.4125
1.6404 HDO
1.92
1.01
1.95
1.93
0.96
2.00
2.02
3.02
3.00
f1 (ppm)

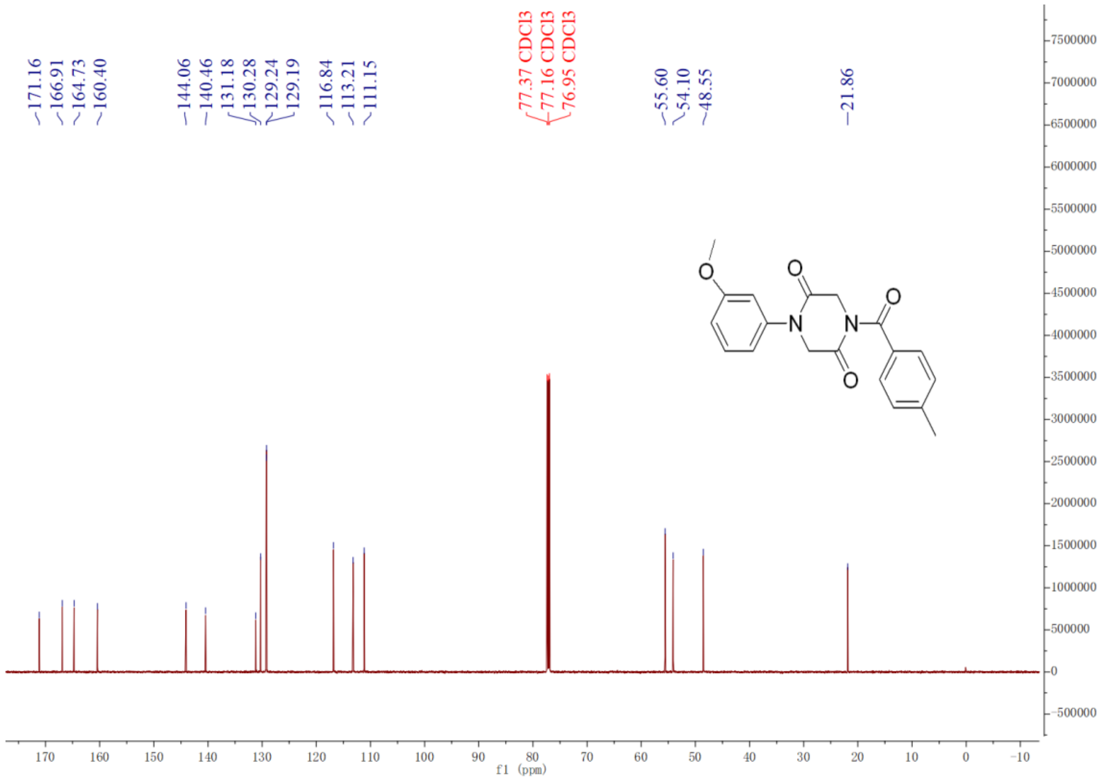
171.16
166.91
164.73
160.40
144.06
140.46
131.18
130.28
129.24
129.19
116.84
113.21
111.15
77.37 CDCl3
77.16 CDCl3
76.95 CDCl3
55.60
54.10
48.55
21.86
f1 (ppm)

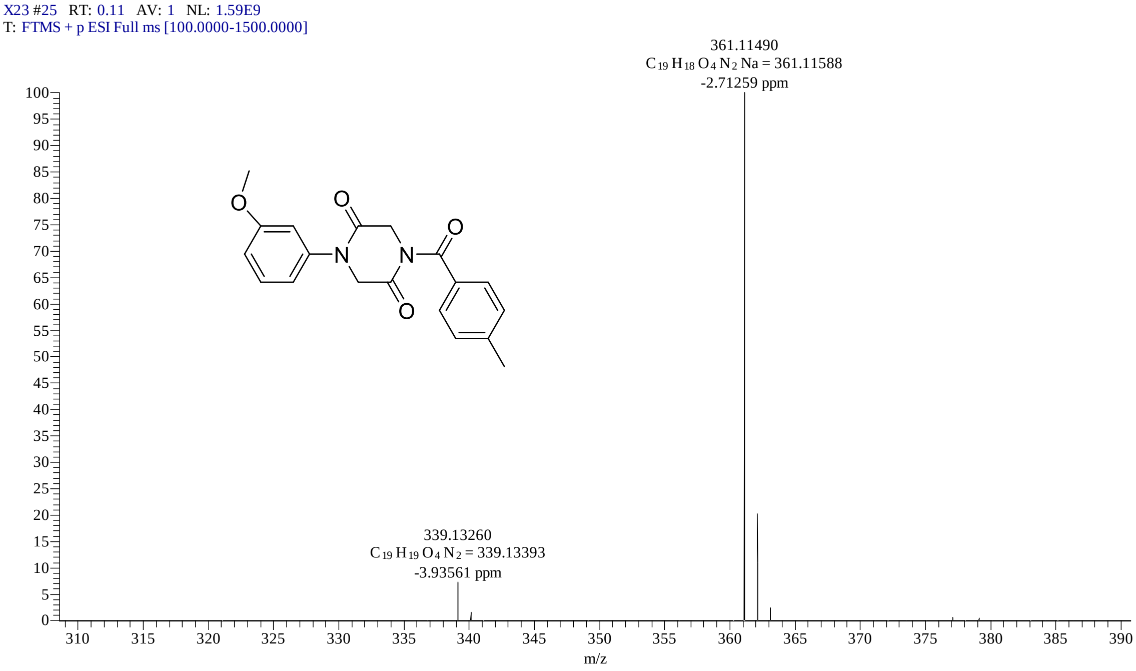

*1-(4-methoxyphenyl)-4-(4-methylbenzoyl)piperazine-2,5-dione (**9f**)*

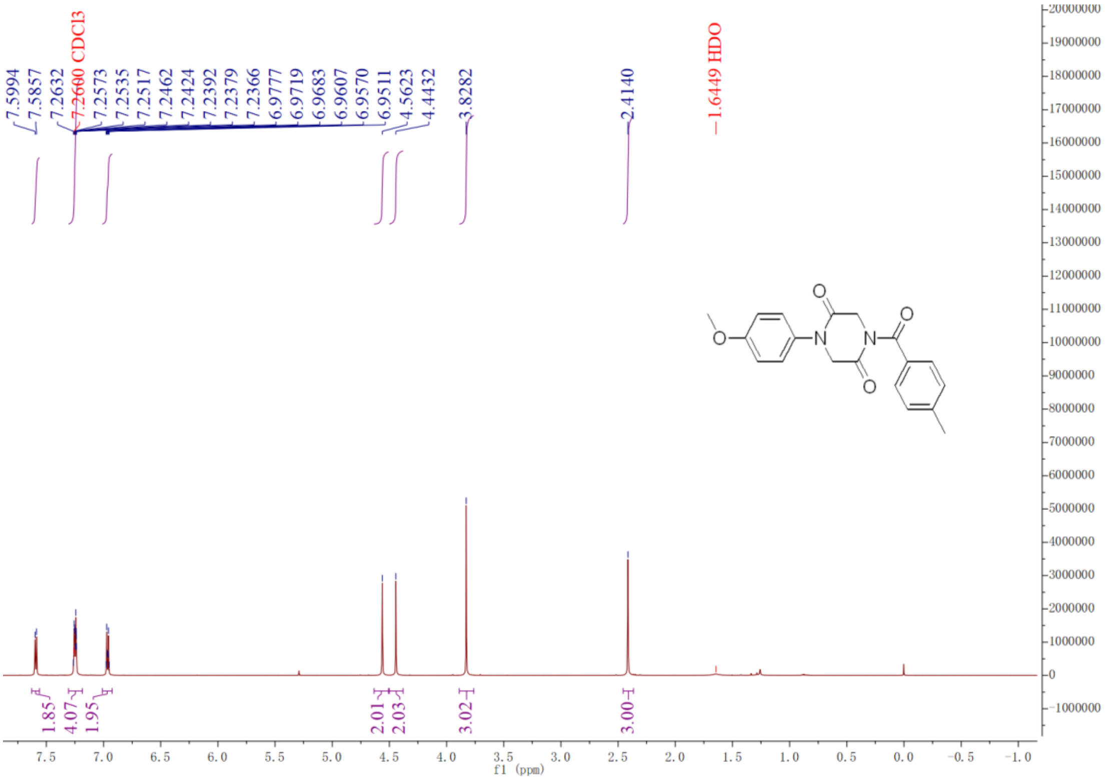

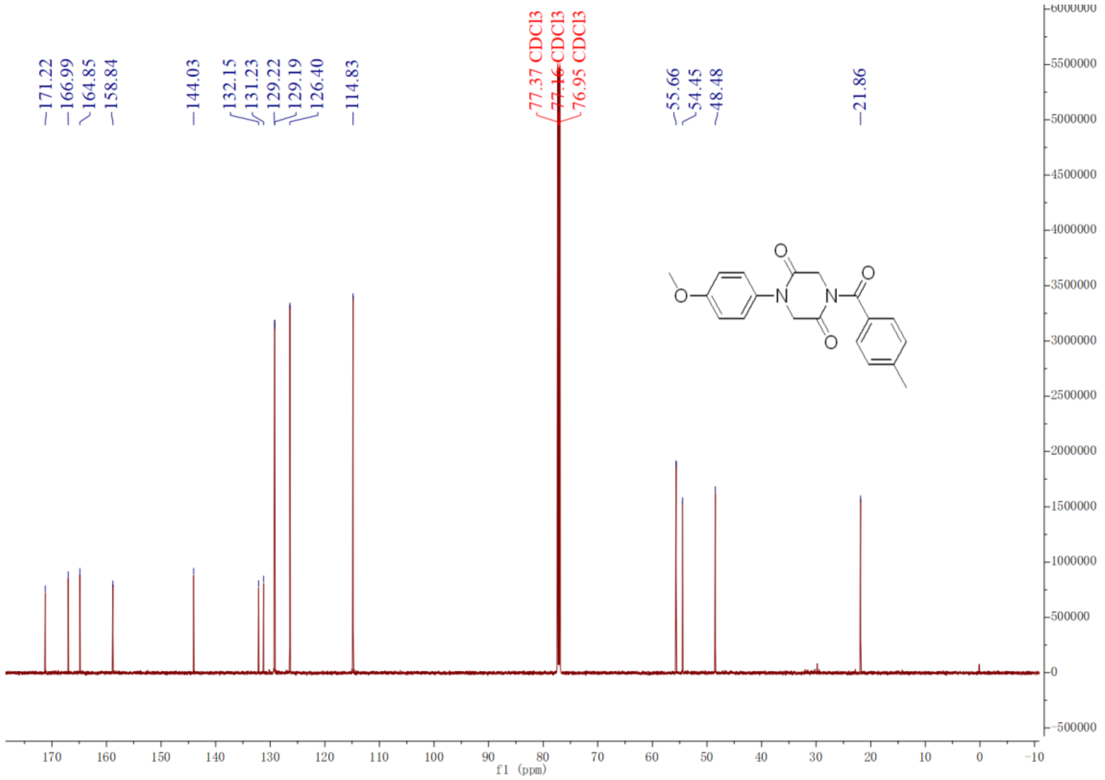

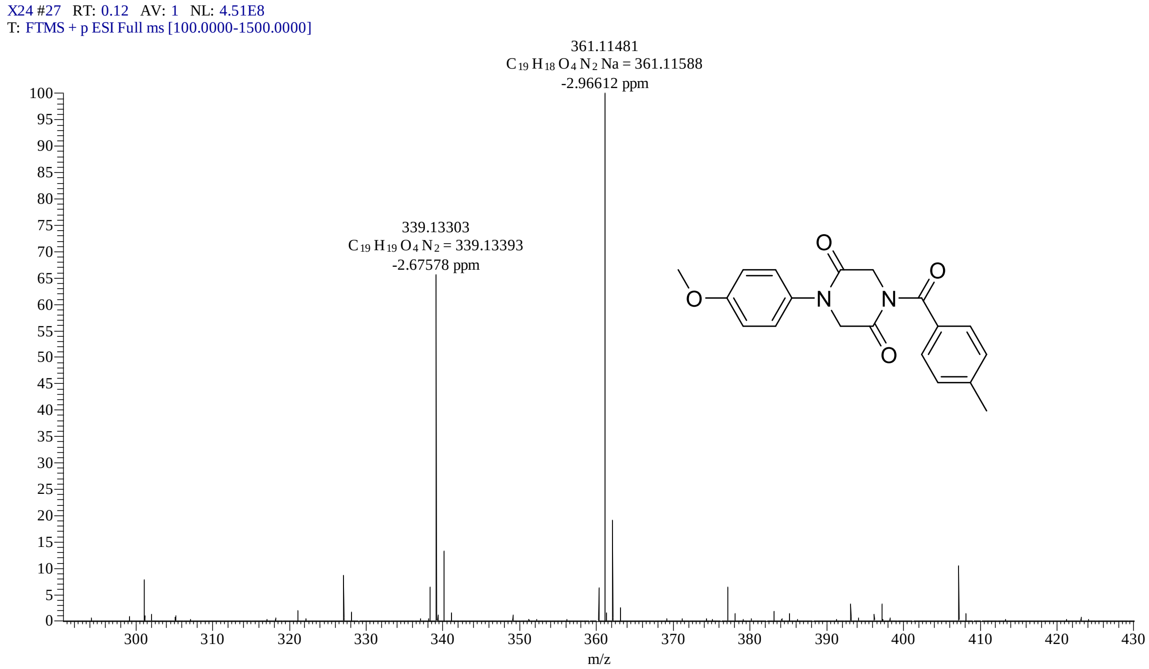

*1-(3,5-dimethoxyphenyl)-4-benzoylpiperazine-2,5-dione (**9g**)*

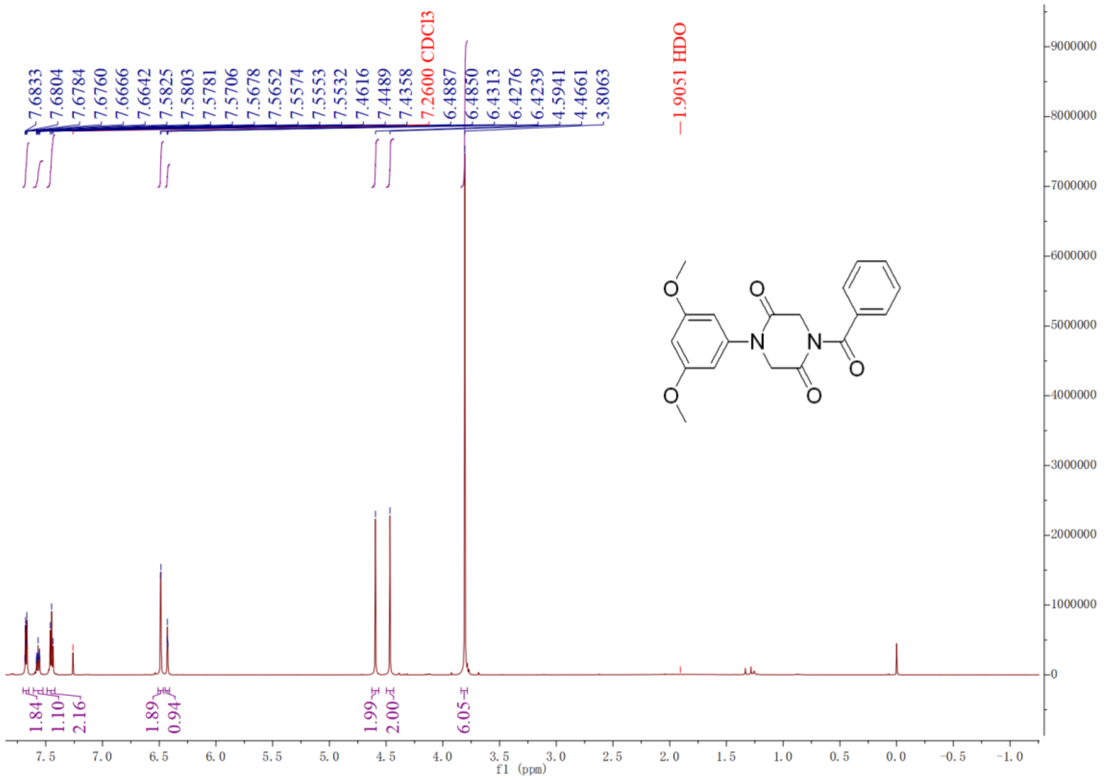
7.6833
7.6804
7.6784
7.6760
7.6666
7.6642
7.5825
7.5803
7.5781
7.5706
7.5678
7.5652
7.5574
7.5553
7.5532
7.4616
7.4489
7.4358
7.2600 CDCl3
6.4887
6.4850
6.4313
6.4276
6.4239
4.5941
4.4661
3.8063
1.9051 HDO
1.84
1.10
2.16
1.89
0.94
1.99
2.00
6.05
O
N
f1 (ppm)

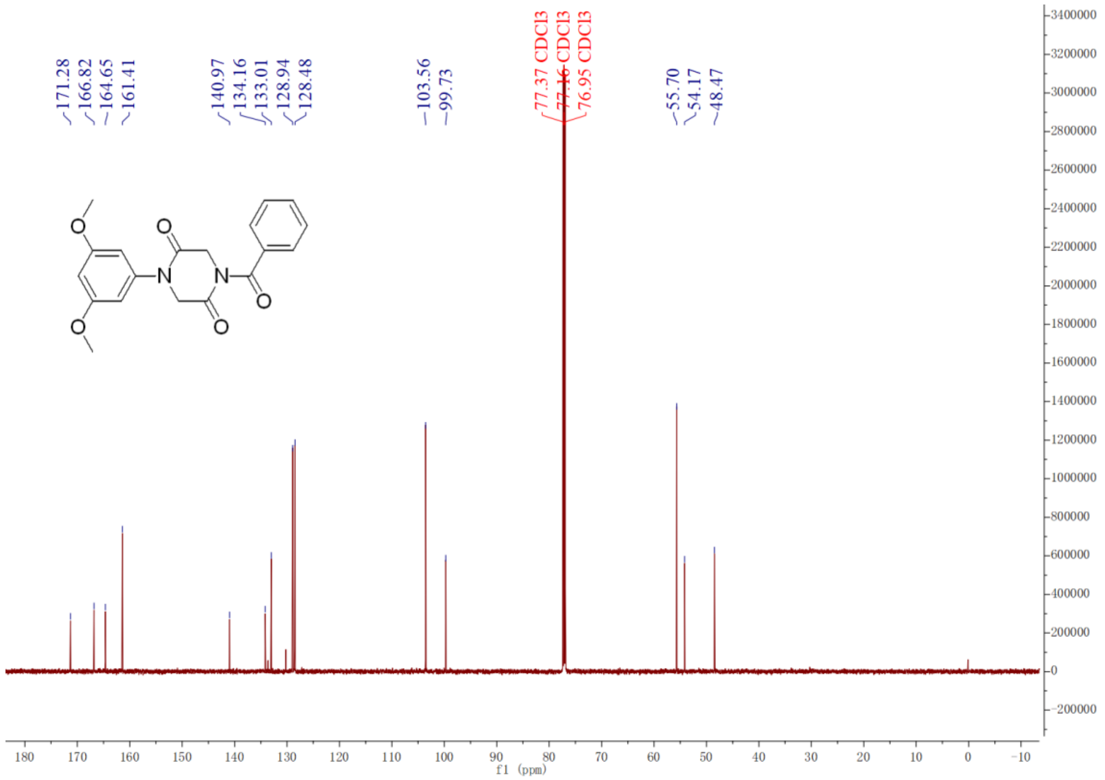
171.28
166.82
164.65
161.41
140.97
134.16
133.01
128.94
128.48
103.56
99.73
77.37 CDCl3
77.16 CDCl3
76.95 CDCl3
55.70
54.17
48.47
O
N
f1 (ppm)

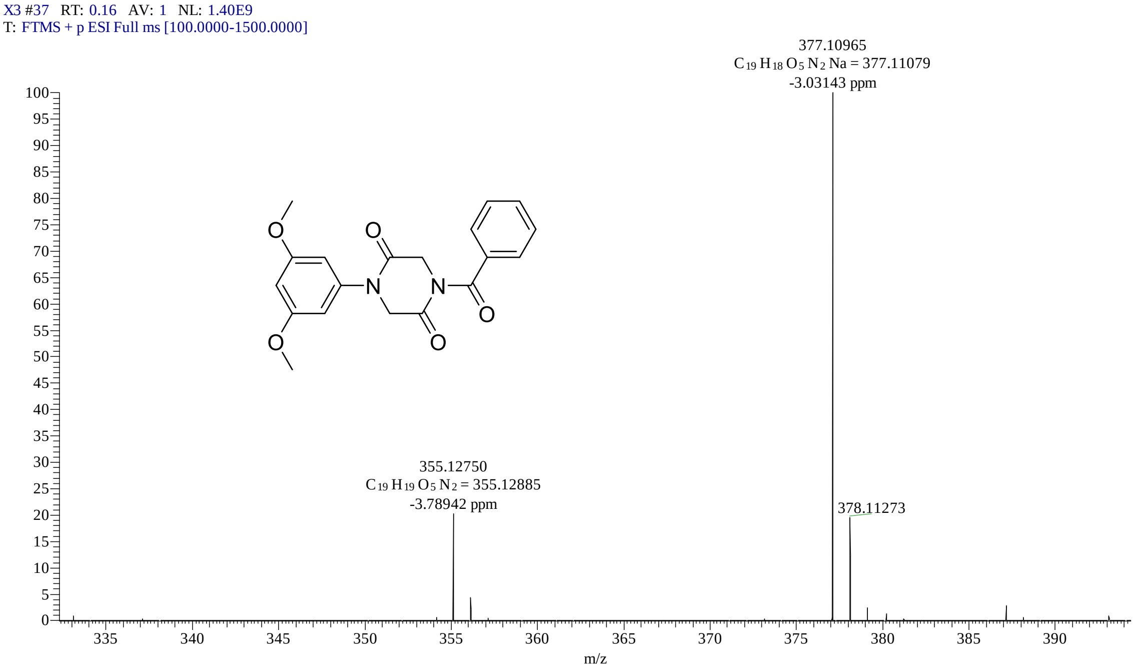

*1-(3,5-dimethoxyphenyl)-4-(4-fluorobenzoyl)piperazine-2,5-dione (**9h**)*

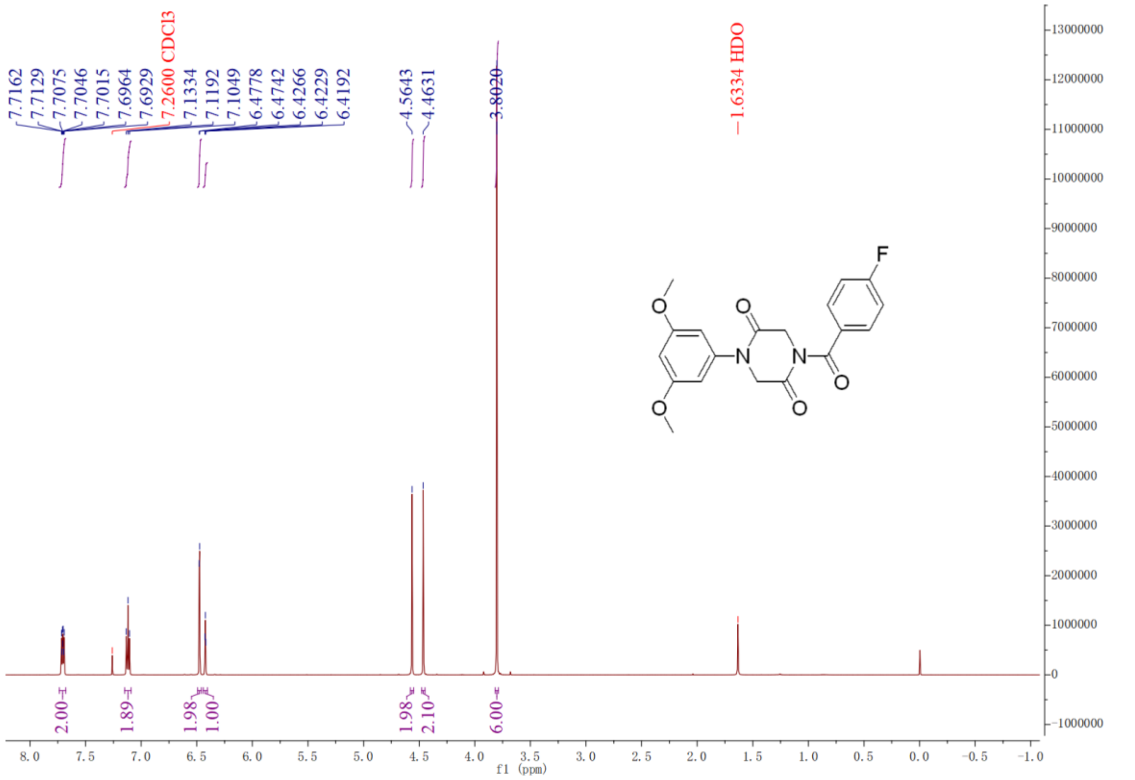

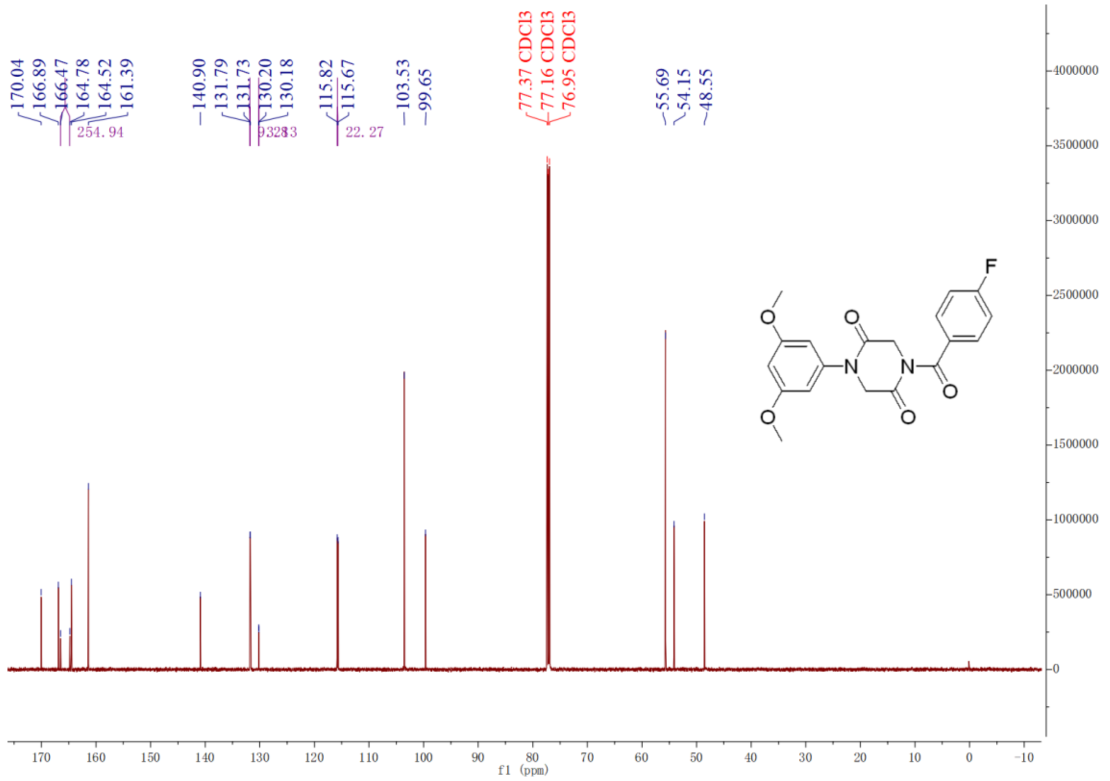
170.04
166.89
166.47
164.78
164.52
161.39
254.94
140.90
131.79
131.73
130.20
130.18
115.82
115.67
22.27
103.53
99.65
77.37 CDCl3
77.16 CDCl3
76.95 CDCl3
55.69
54.15
48.55
f1 (ppm)
F
O
N

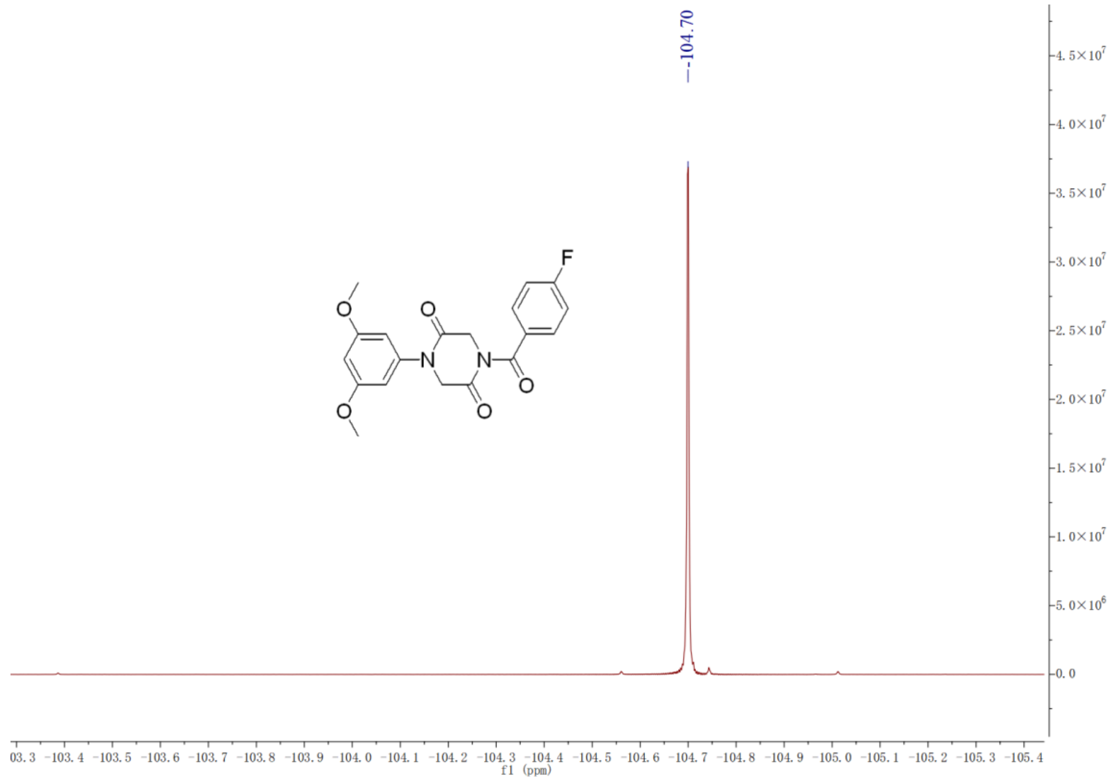
-104.70
f1 (ppm)
F
O
N

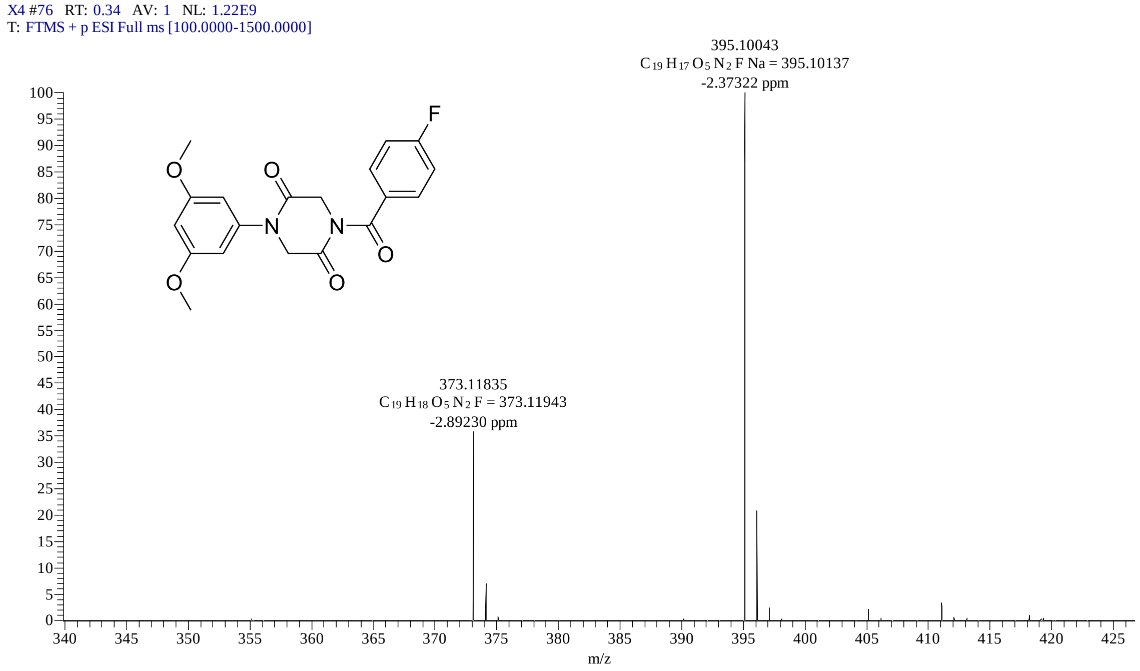

*1-(3,5-dimethoxyphenyl)-4-(4-(trifluoromethyl)benzoyl)piperazine-2,5-dione (**9i**)*

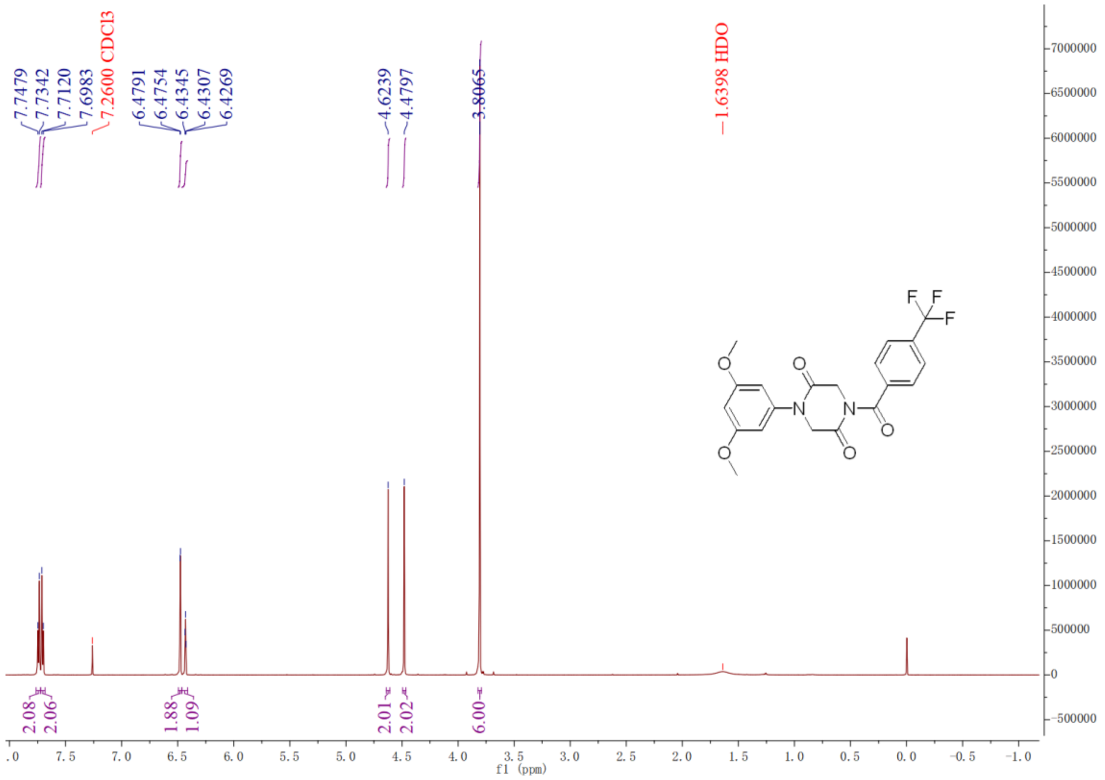

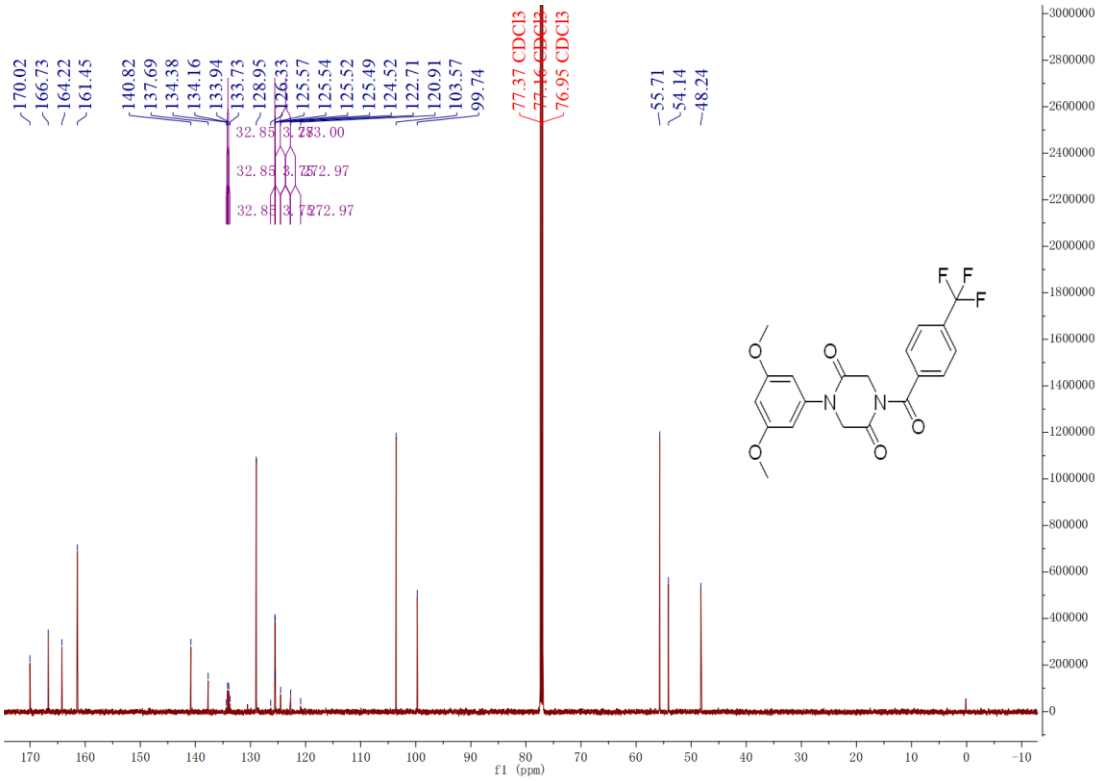
170.02
166.73
164.22
161.45
140.82
137.69
134.38
134.16
133.94
133.73
128.95
126.33
125.57
125.54
125.52
125.49
124.52
122.71
120.91
103.57
99.74
77.37 CDCl3
77.16 CDCl3
76.95 CDCl3
55.71
54.14
48.24
f1 (ppm)

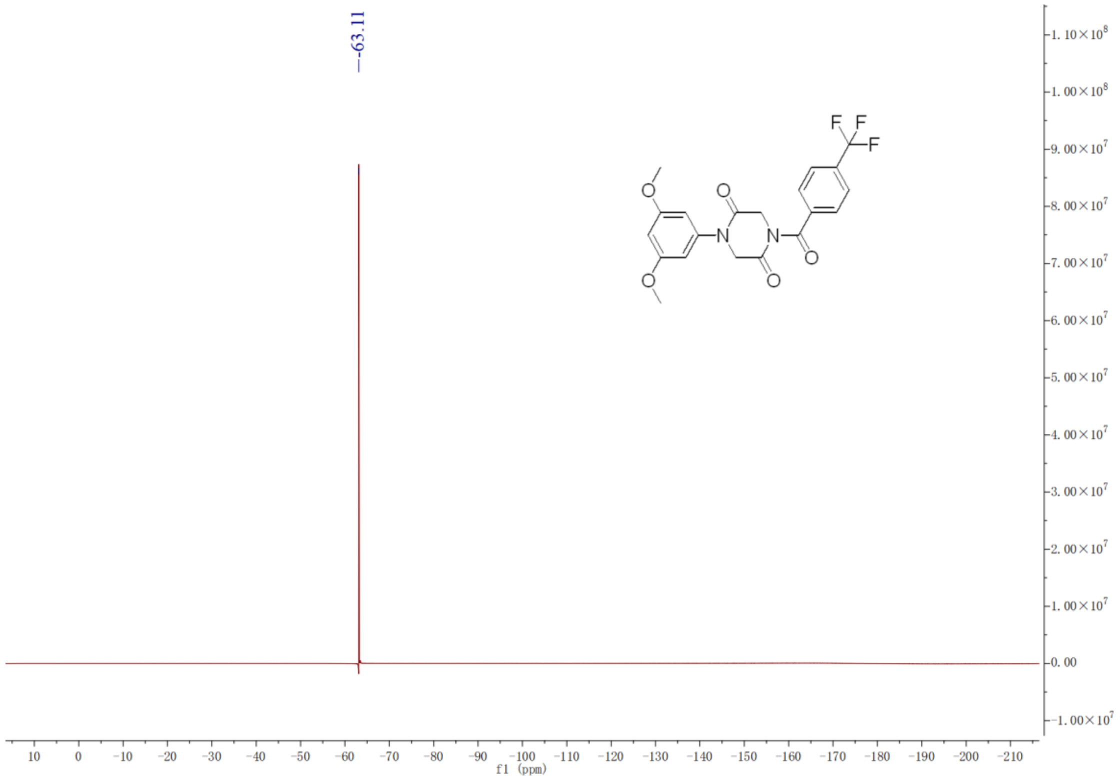
-63.11
f1 (ppm)

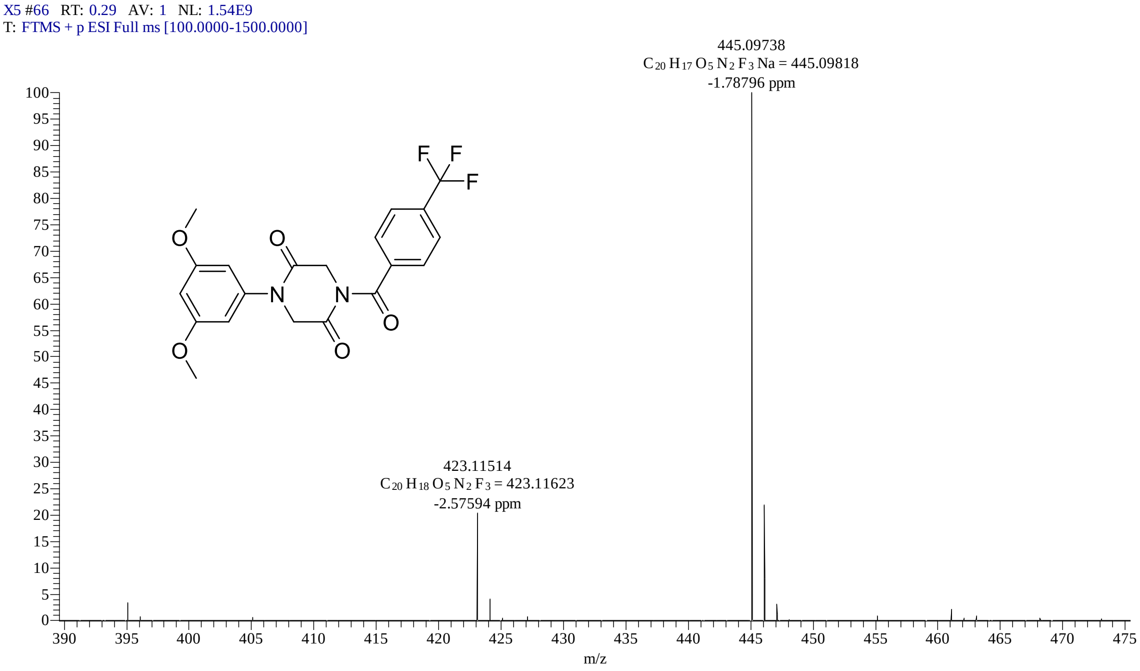

*1-(3,5-dimethoxyphenyl)-4-(4-(tert-butyl)benzoyl)piperazine-2,5-dione (**9j**)*

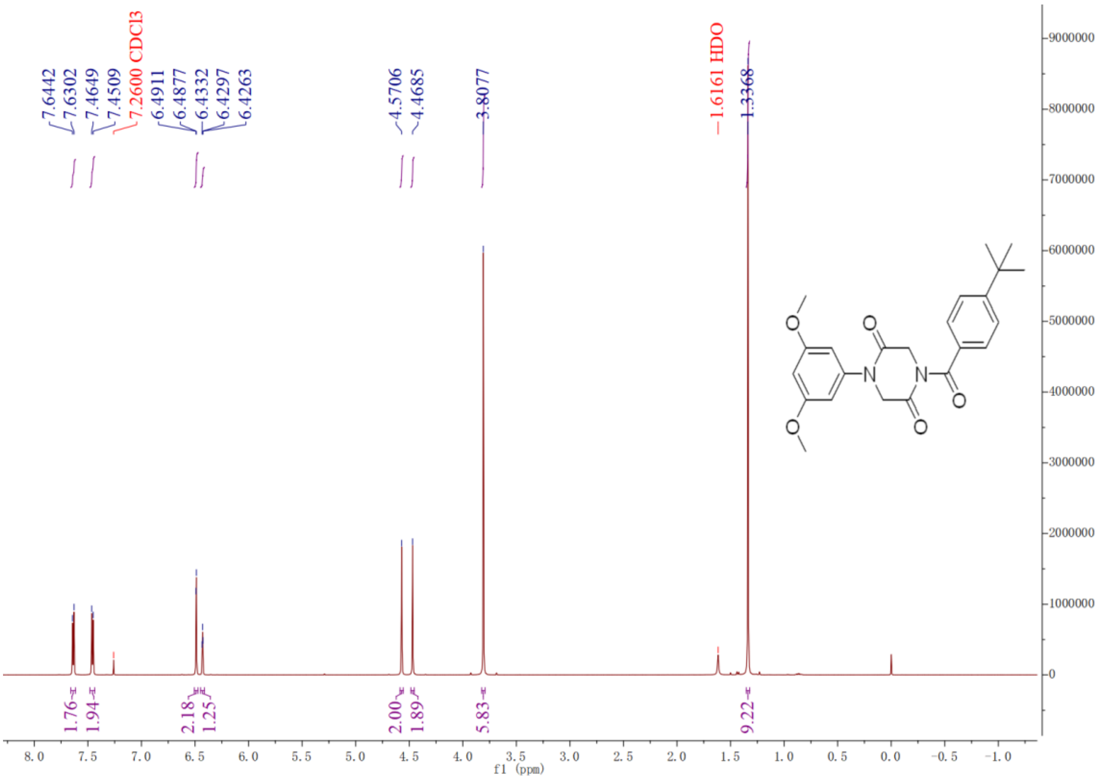

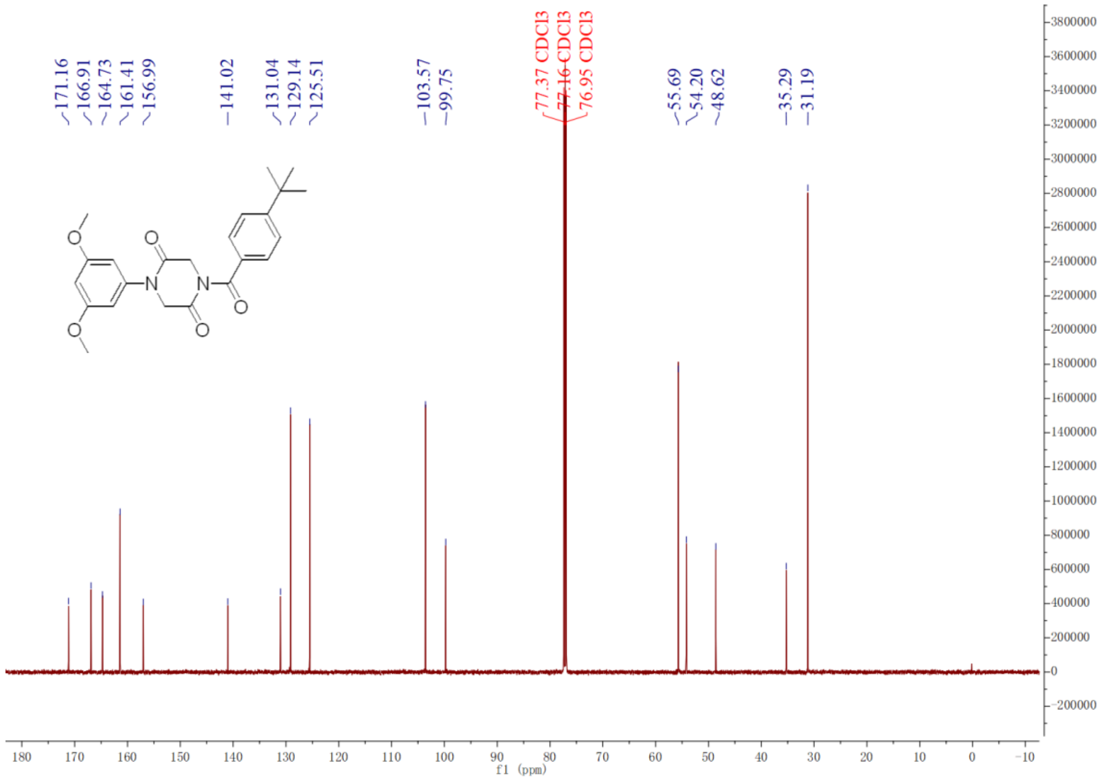

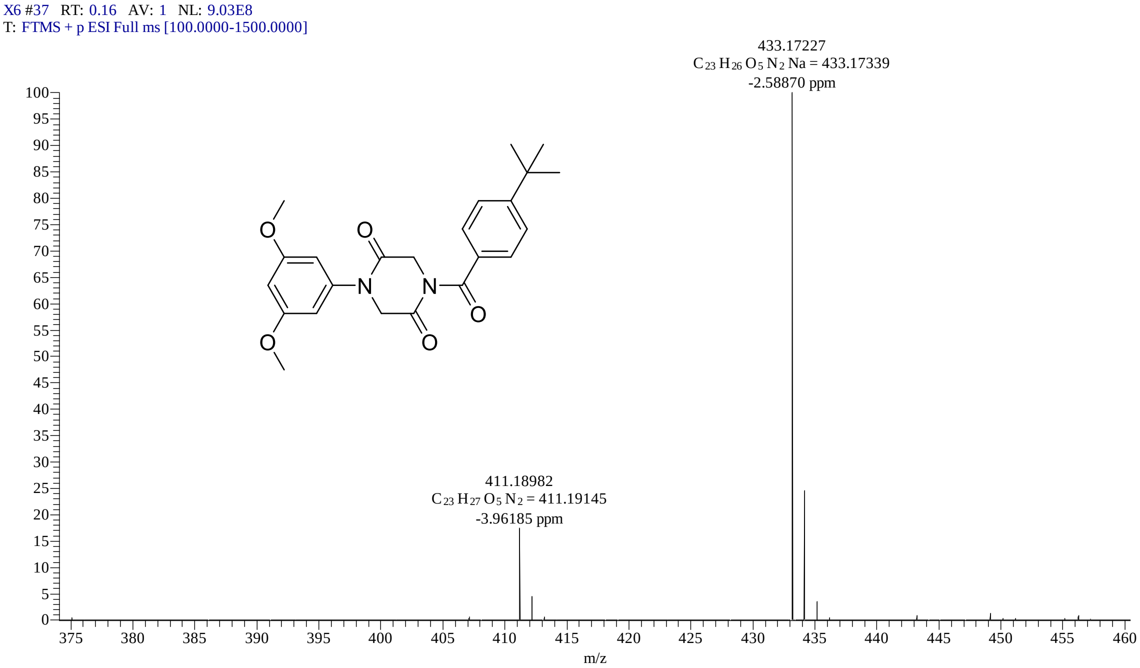

1-(3,5-dimethoxyphenyl)-4-acetylpiperazine-2,5-dione ***(9k)***

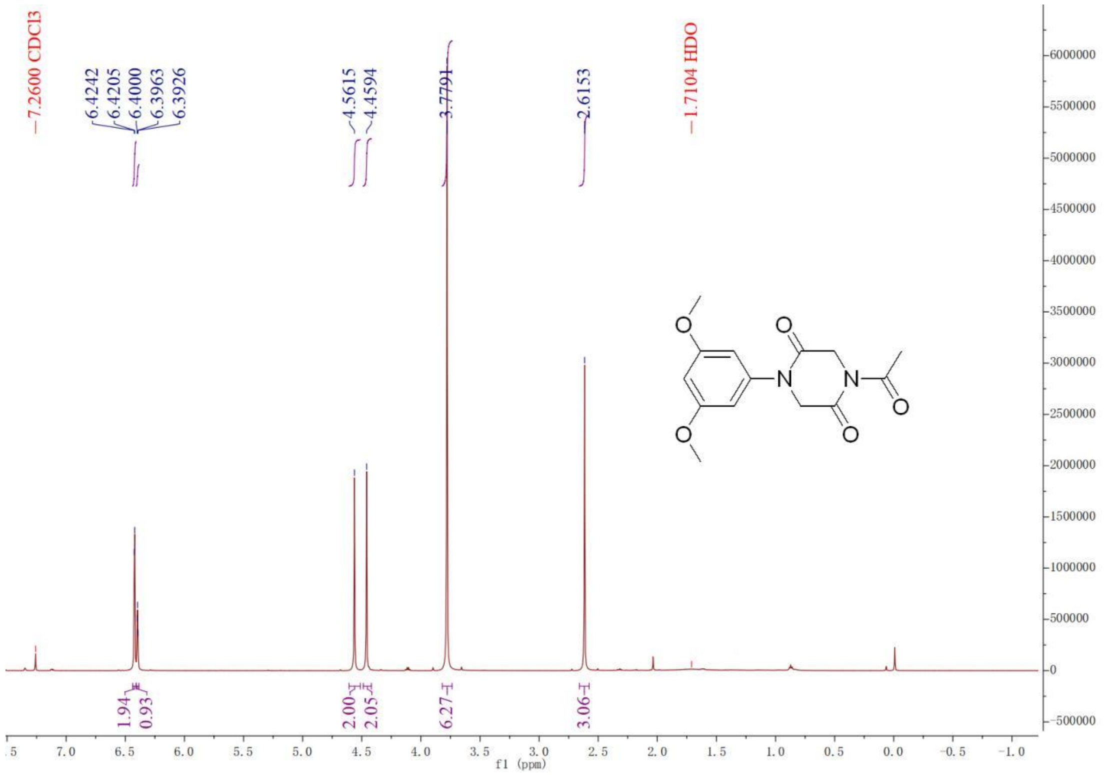

7.2600 CDCl3
6.4242
6.4205
6.4000
6.3963
6.3926
4.5615
4.4594
3.7791
2.6153
1.7104 HDO
1.94
0.93
2.00
2.05
6.27
3.06
f1 (ppm)

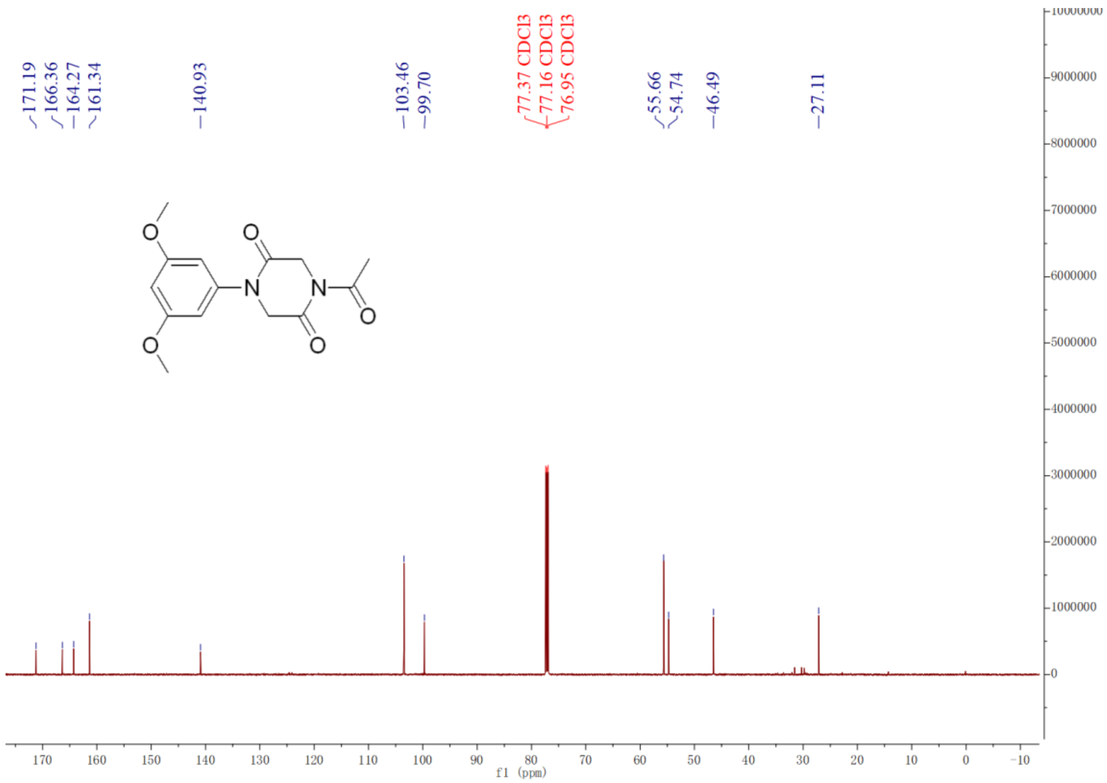

171.19
166.36
164.27
161.34
140.93
103.46
99.70
77.37 CDCl3
77.16 CDCl3
76.95 CDCl3
55.66
54.74
46.49
27.11
f1 (ppm)

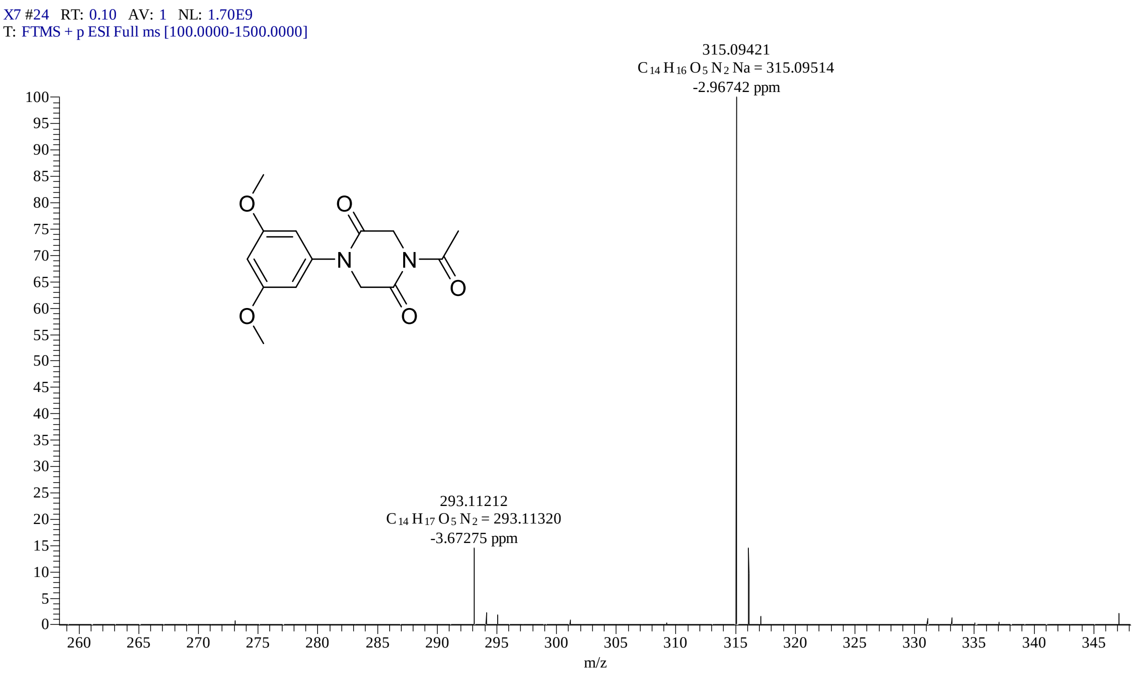

*1-(3,5-dimethoxyphenyl)-4-(3-bromobenzoyl)piperazine-2,5-dione (**9l**)*

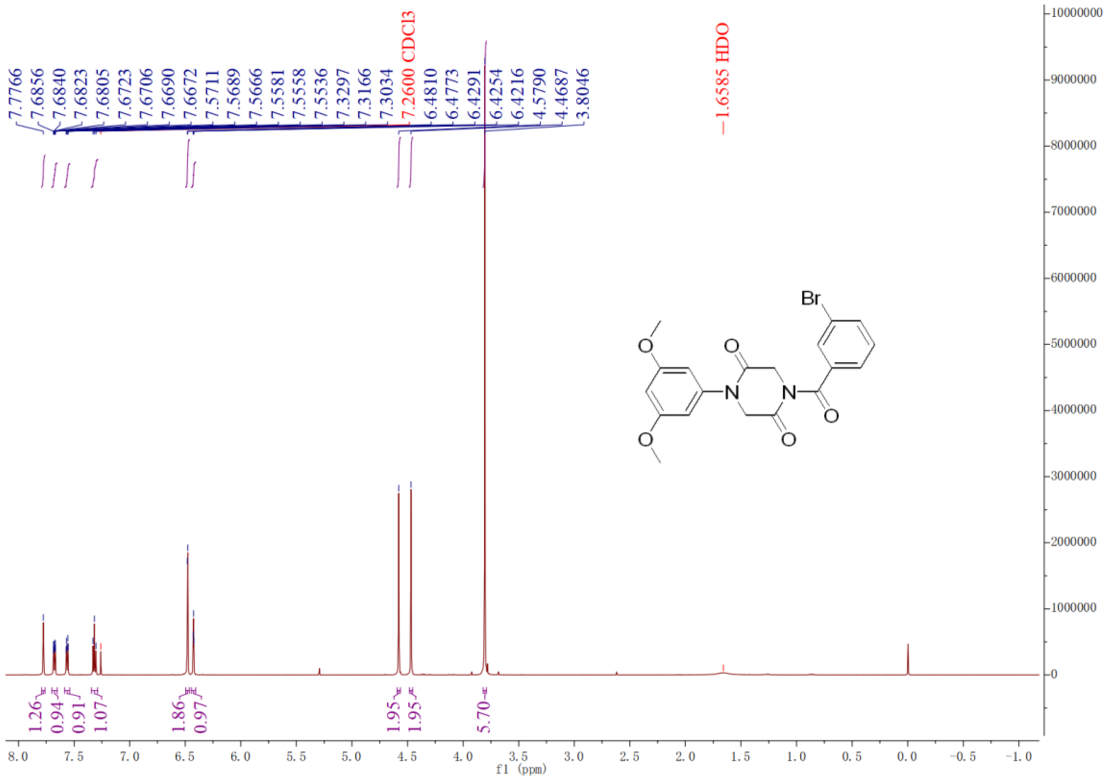

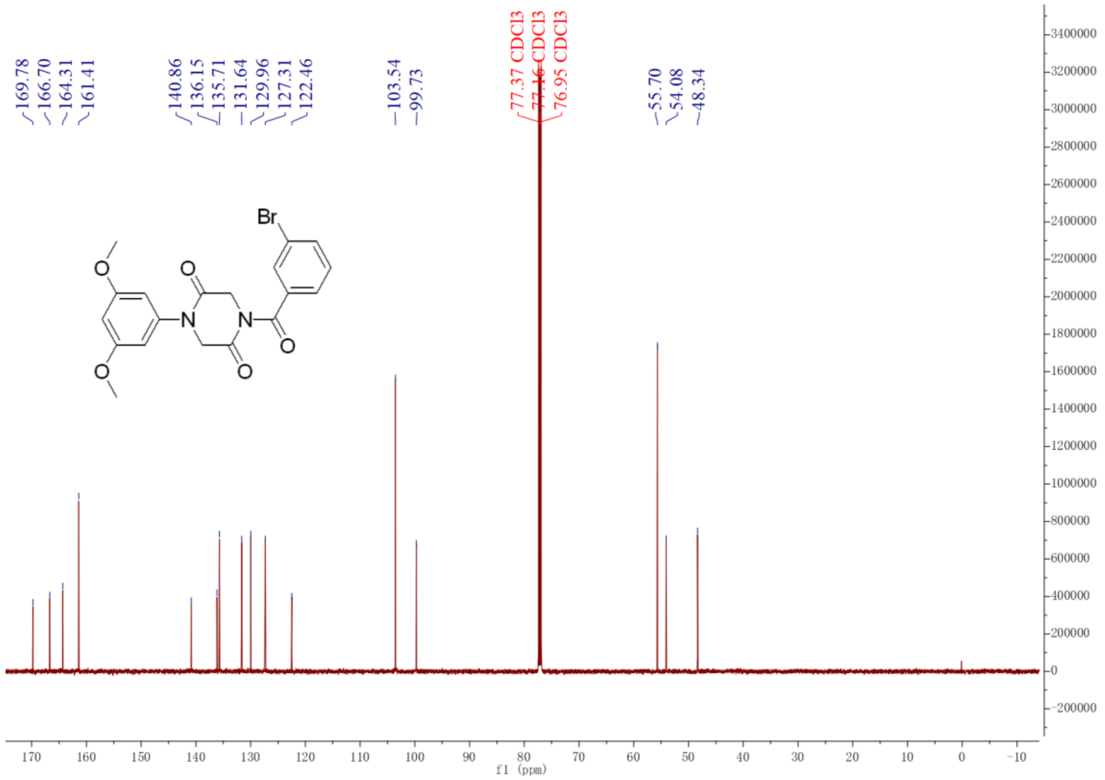

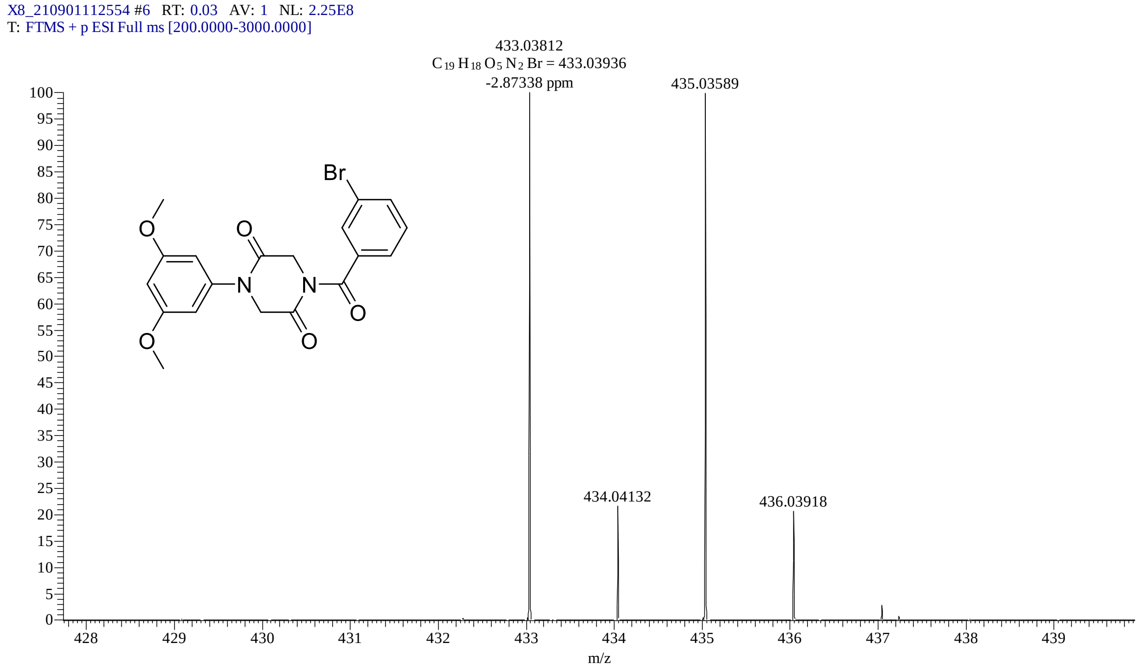

1-(3,5-dimethoxyphenyl)-4-propionylpiperazine-2,5-dione (***9m***)

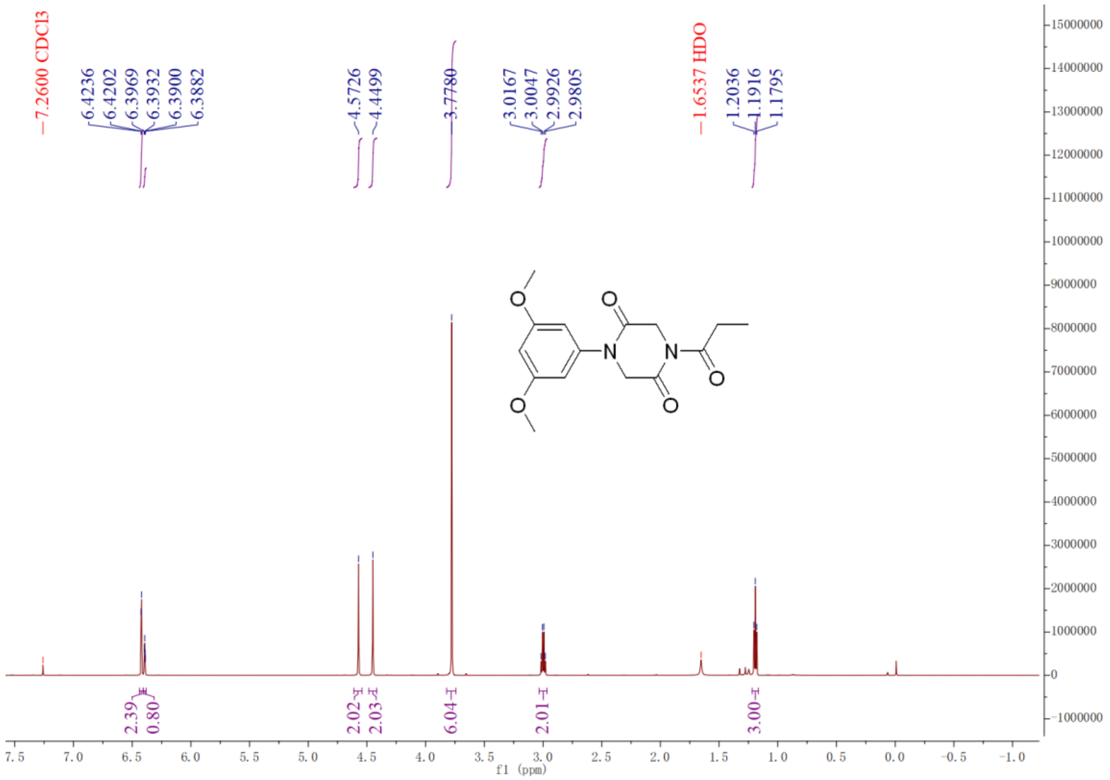
7.2600 CDCl3
6.4236
6.4202
6.3969
6.3932
6.3900
6.3882
4.5726
4.4499
3.7780
3.0167
3.0047
2.9926
2.9805
1.6537 HDO
1.2036
1.1916
1.1795
2.39
0.80
2.02
2.03
6.04
2.01
3.00
f1 (ppm)

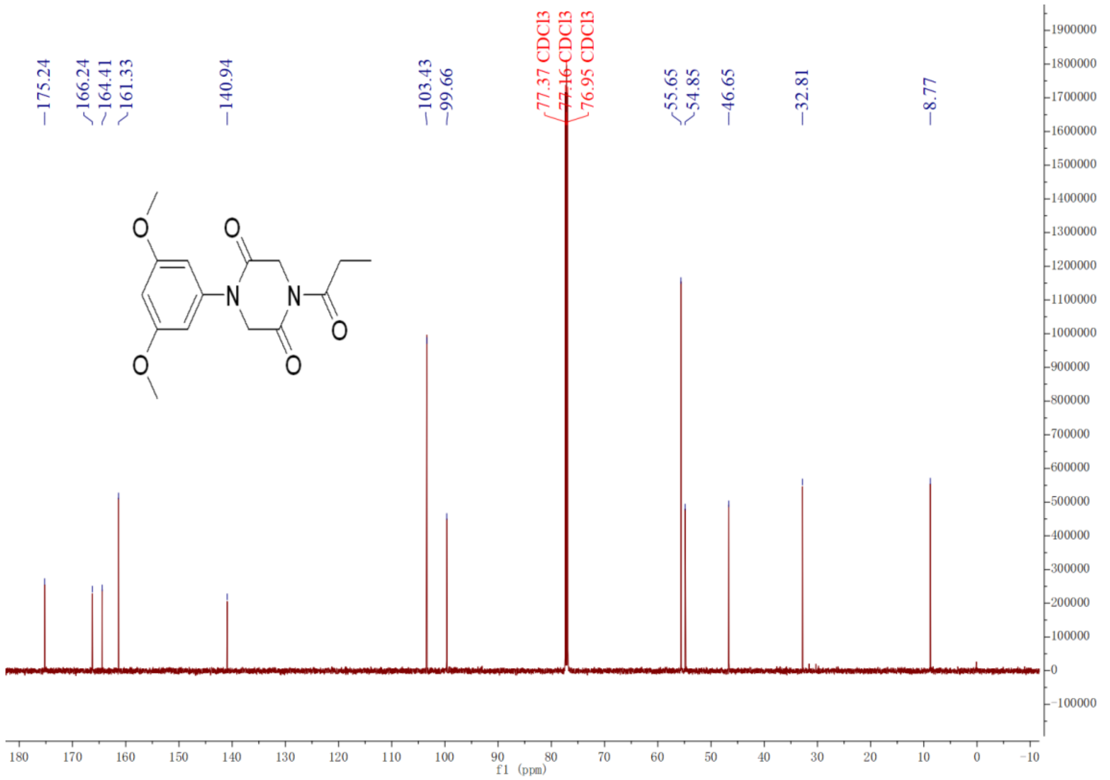
175.24
166.24
164.41
161.33
140.94
103.43
99.66
77.37 CDCl3
77.16 CDCl3
76.95 CDCl3
55.65
54.85
46.65
32.81
8.77
f1 (ppm)

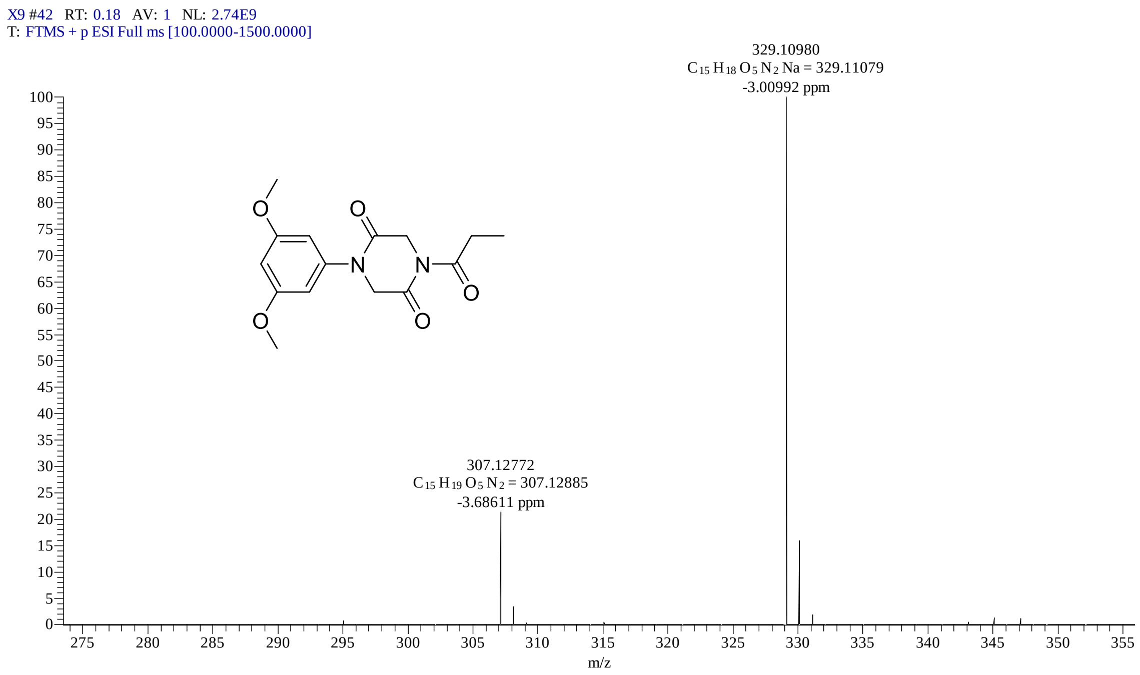

*1-(3,5-dimethoxyphenyl)-4-butyrylpiperazine-2,5-dione (**9n**)*

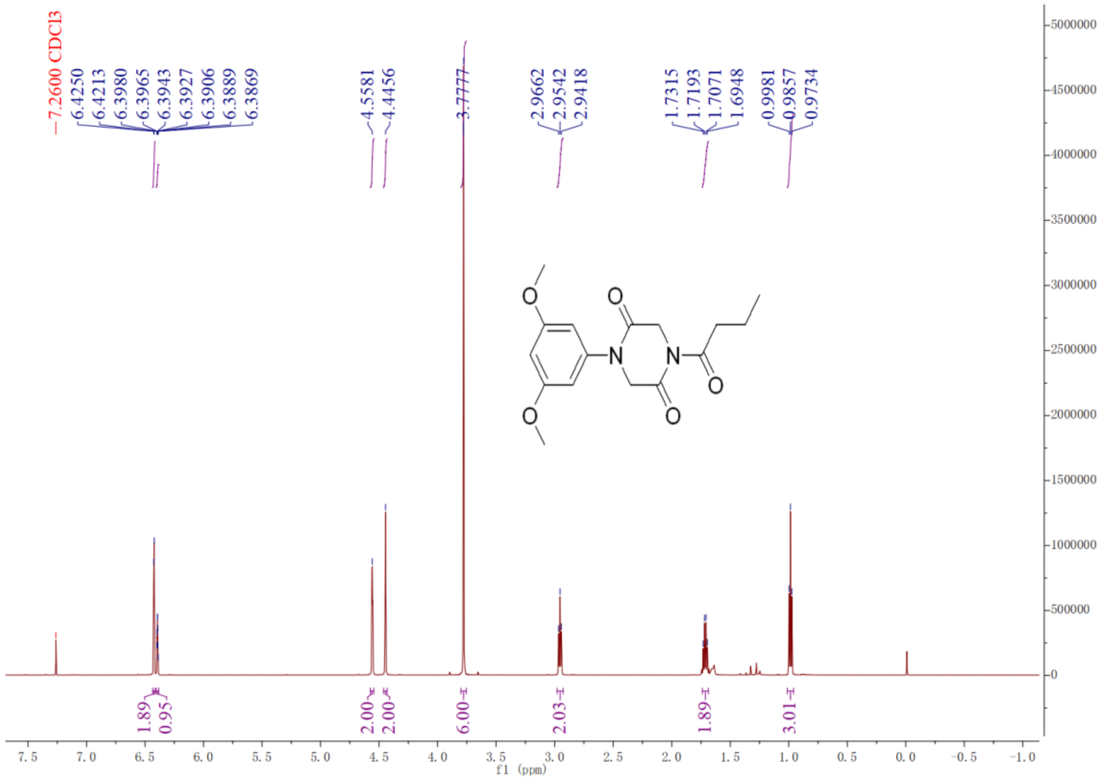

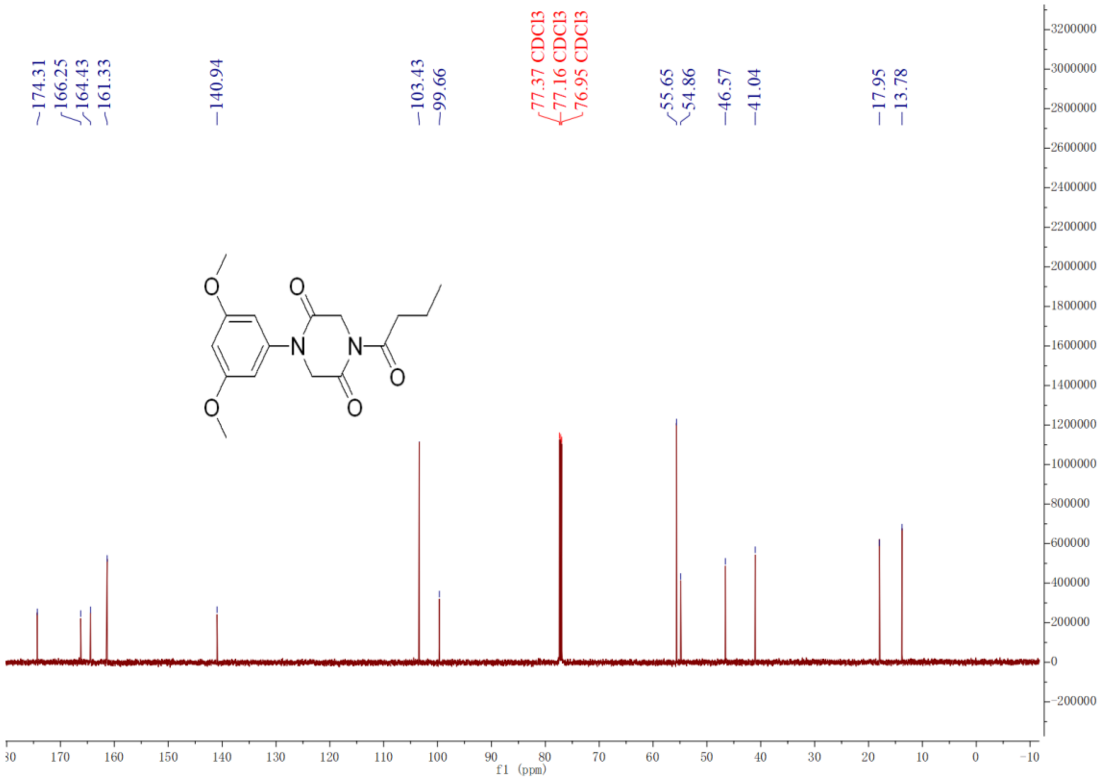

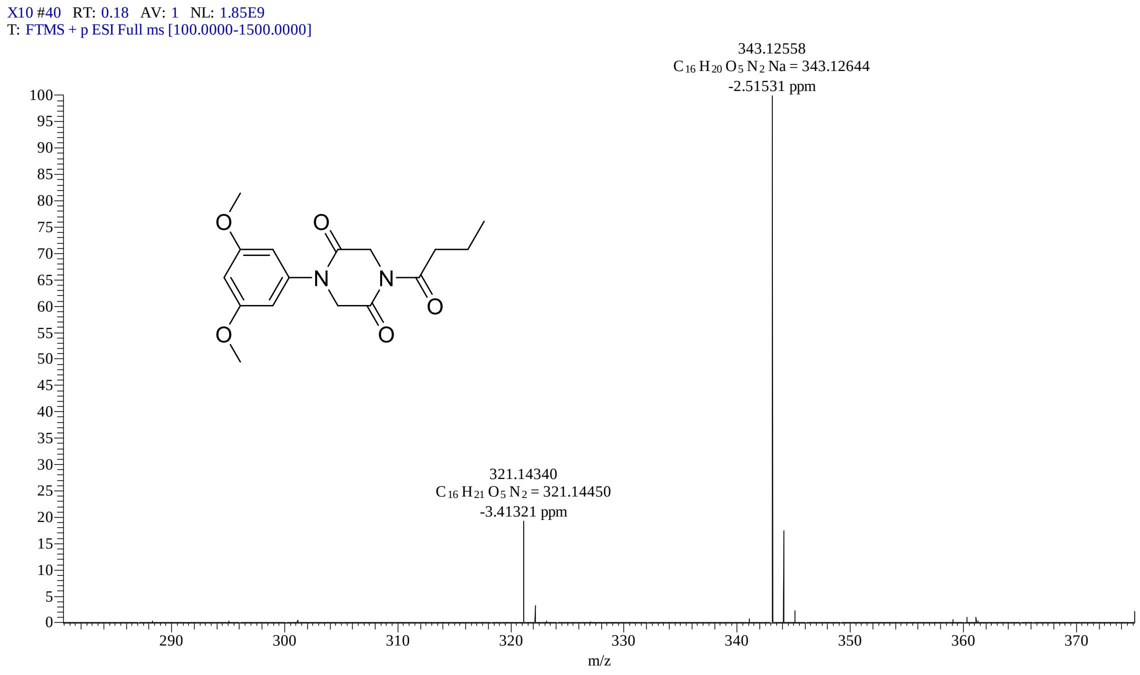

*1-(3,5-difluorobenzoyl)-4-(3,5-dimethoxyphenyl)piperazine-2,5-dione (**9o**)*

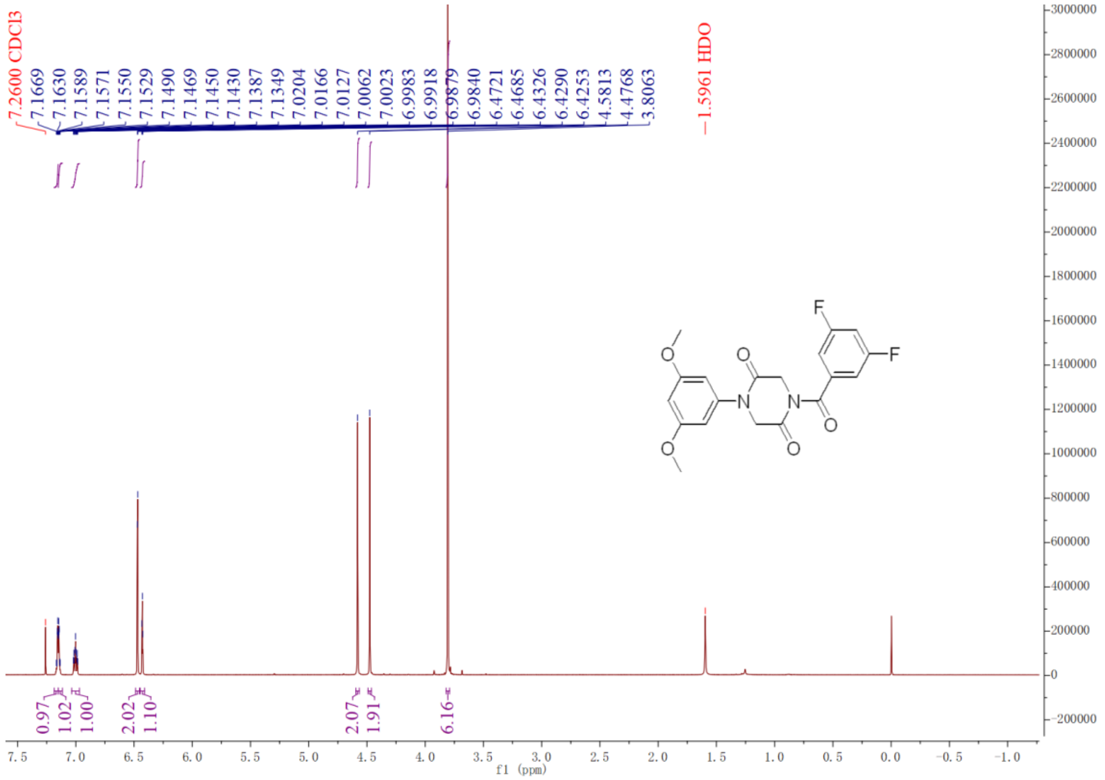
7.2600 CDCl3
7.1669
7.1630
7.1589
7.1571
7.1550
7.1529
7.1490
7.1469
7.1450
7.1430
7.1387
7.1349
7.0204
7.0166
7.0127
7.0062
7.0023
6.9983
6.9918
6.9879
6.9840
6.4721
6.4685
6.4326
6.4290
6.4253
4.5813
4.4768
3.8063
1.5961 HDO
0.97
1.02
1.00
2.02
1.10
2.07
1.91
6.16
f1 (ppm)

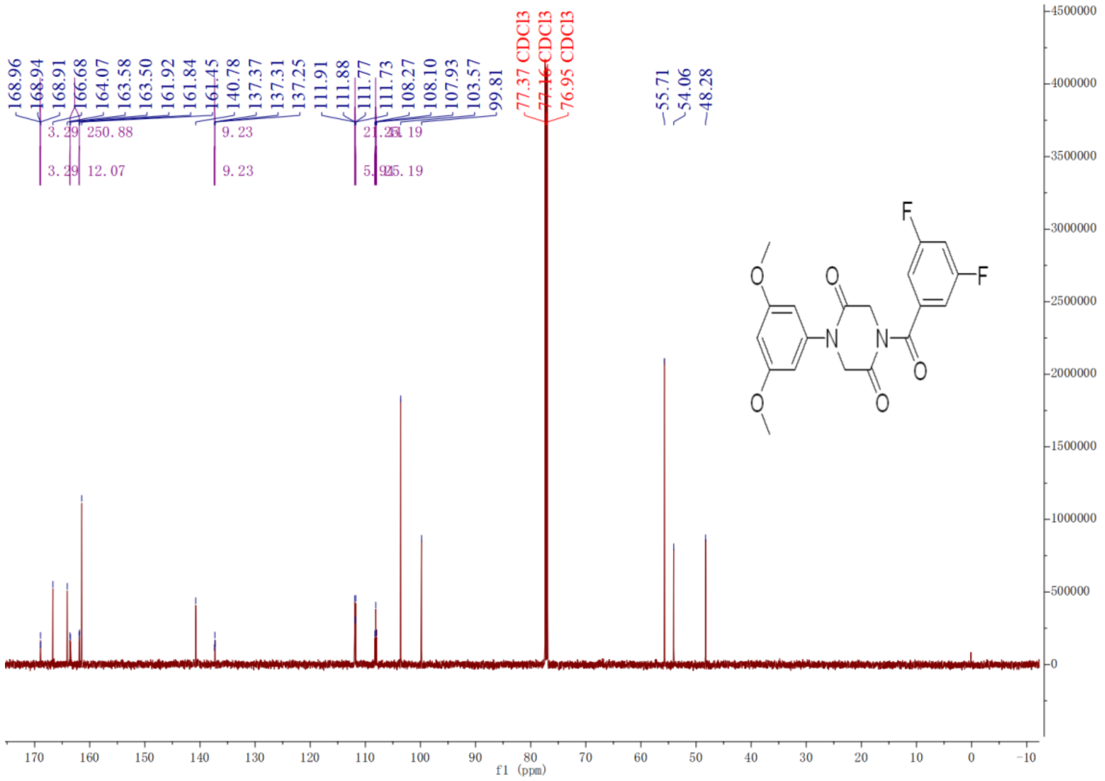
168.96
168.94
168.91
166.68
164.07
163.58
163.50
161.92
161.84
161.45
140.78
137.37
137.31
137.25
111.91
111.88
111.77
111.73
108.27
108.10
107.93
103.57
99.81
77.37 CDCl3
77.16 CDCl3
76.95 CDCl3
55.71
54.06
48.28
3.29
250.88
12.07
9.23
f1 (ppm)

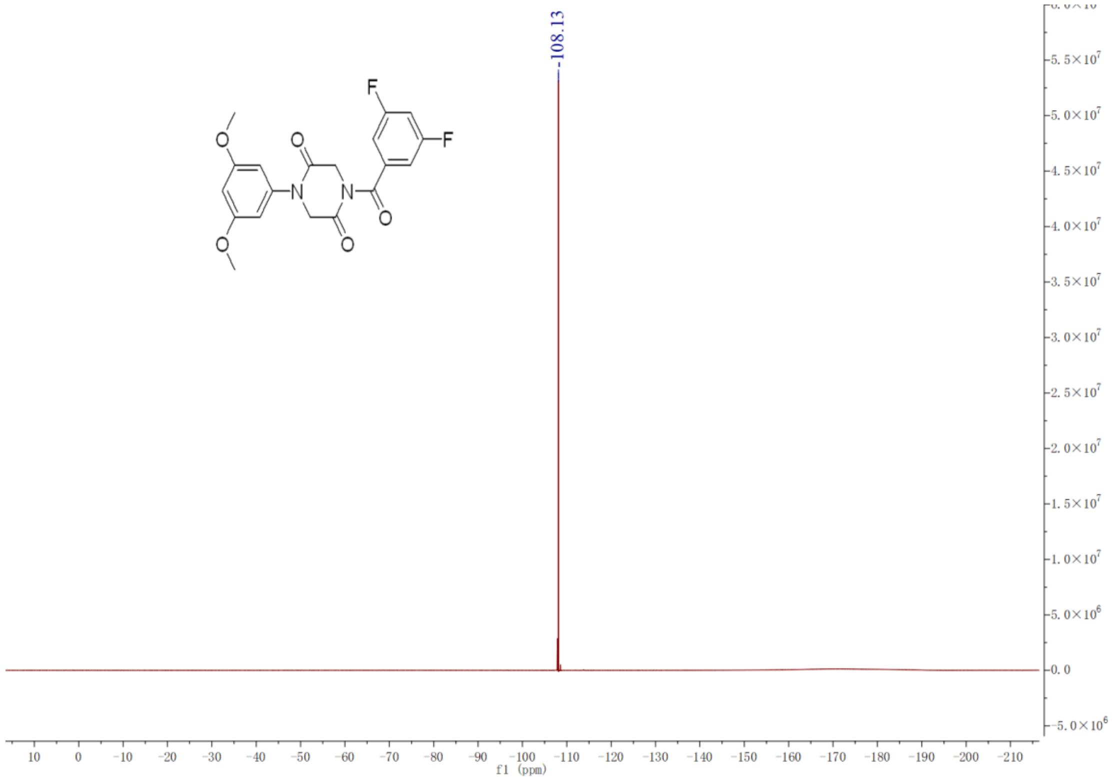

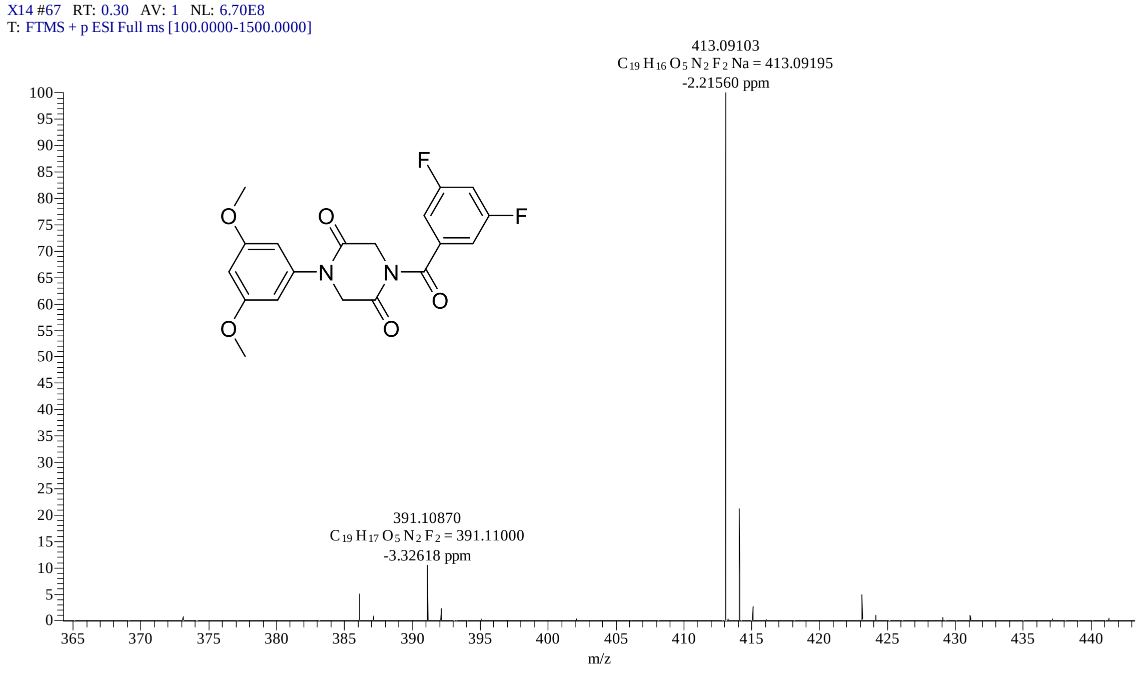

*1-(3,5-dimethoxyphenyl)-4-pivaloylpiperazine-2,5-dione (**9p**)*

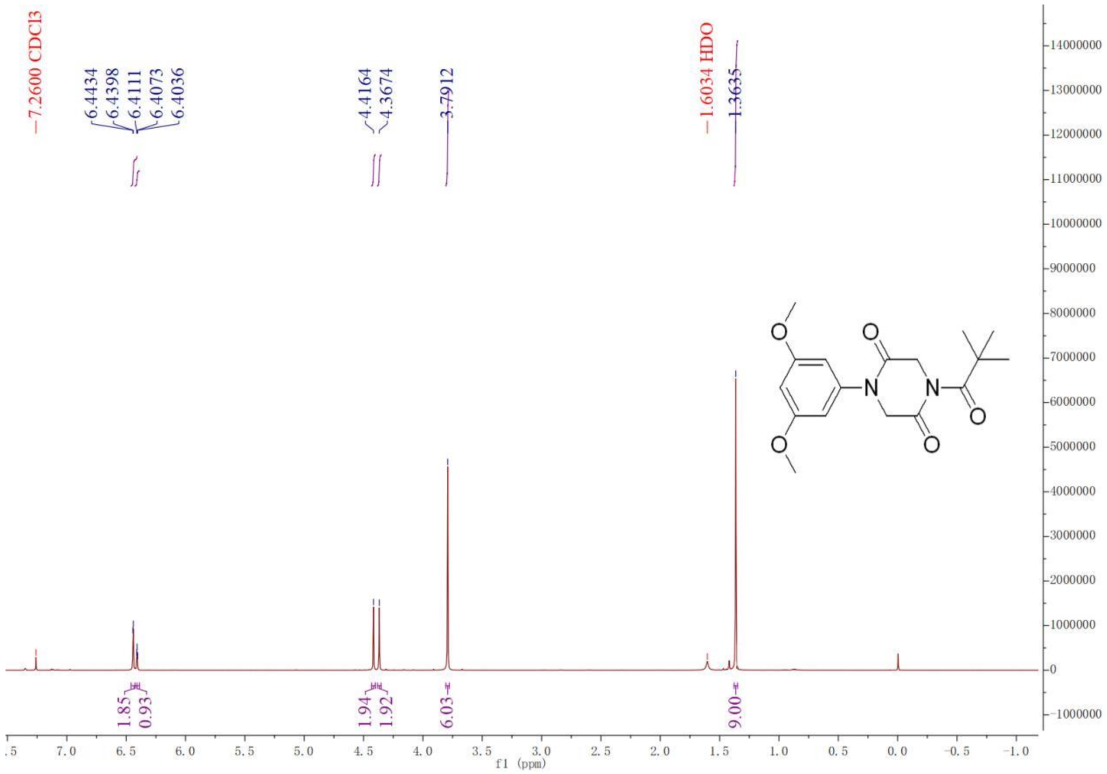
7.2600 CDCl3
6.4434
6.4398
6.4111
6.4073
6.4036
4.4164
4.3674
3.7912
1.6034 HDO
1.3635
1.85
0.93
1.94
1.92
6.03
9.00
f1 (ppm)

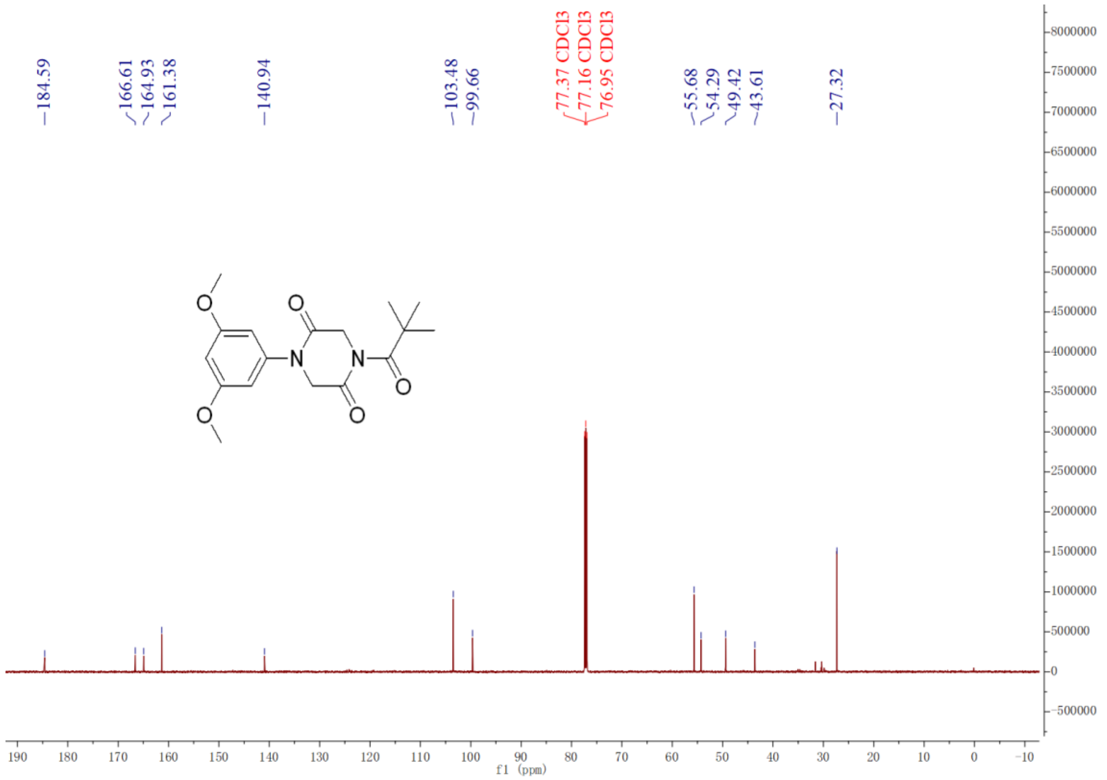
184.59
166.61
164.93
161.38
140.94
103.48
99.66
77.37 CDCl3
77.16 CDCl3
76.95 CDCl3
55.68
54.29
49.42
43.61
27.32
f1 (ppm)

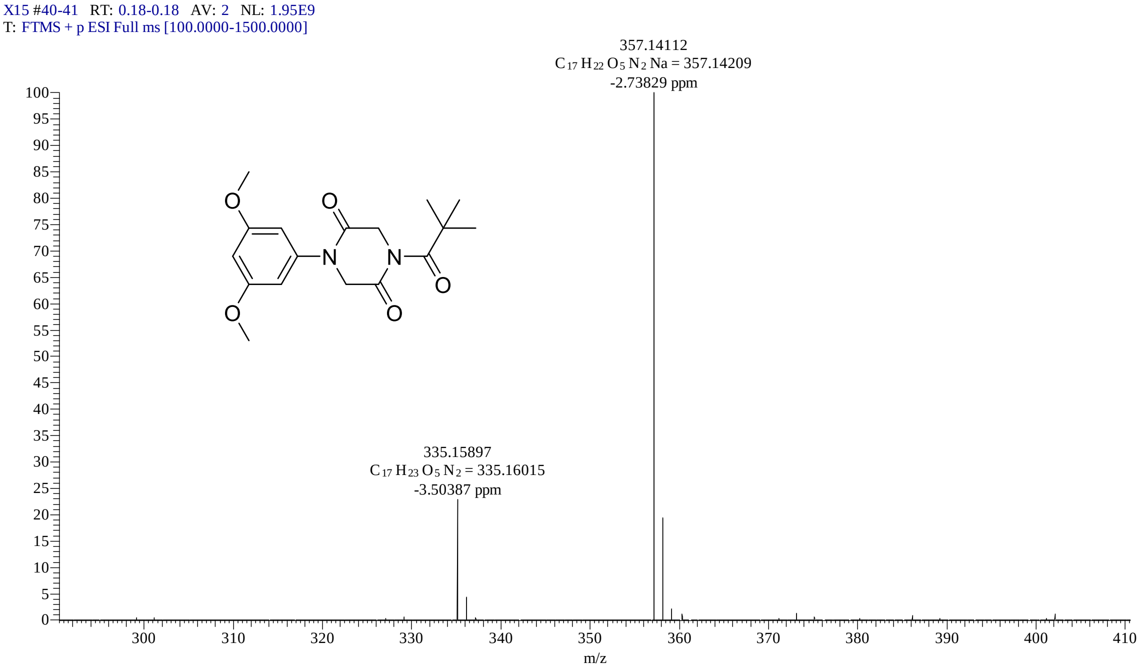

*1-(3,5-dimethoxyphenyl)-4-(cyclopropanecarbonyl)piperazine-2,5-dione (**9q**)*

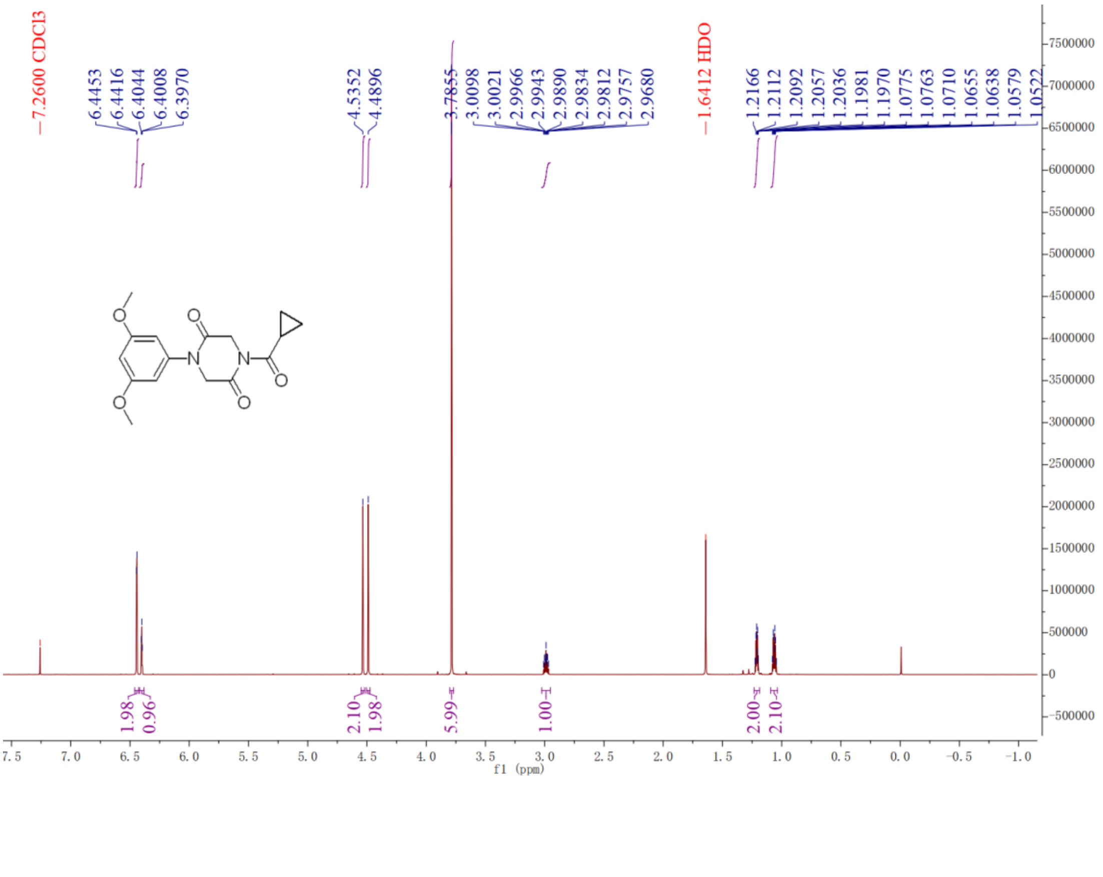

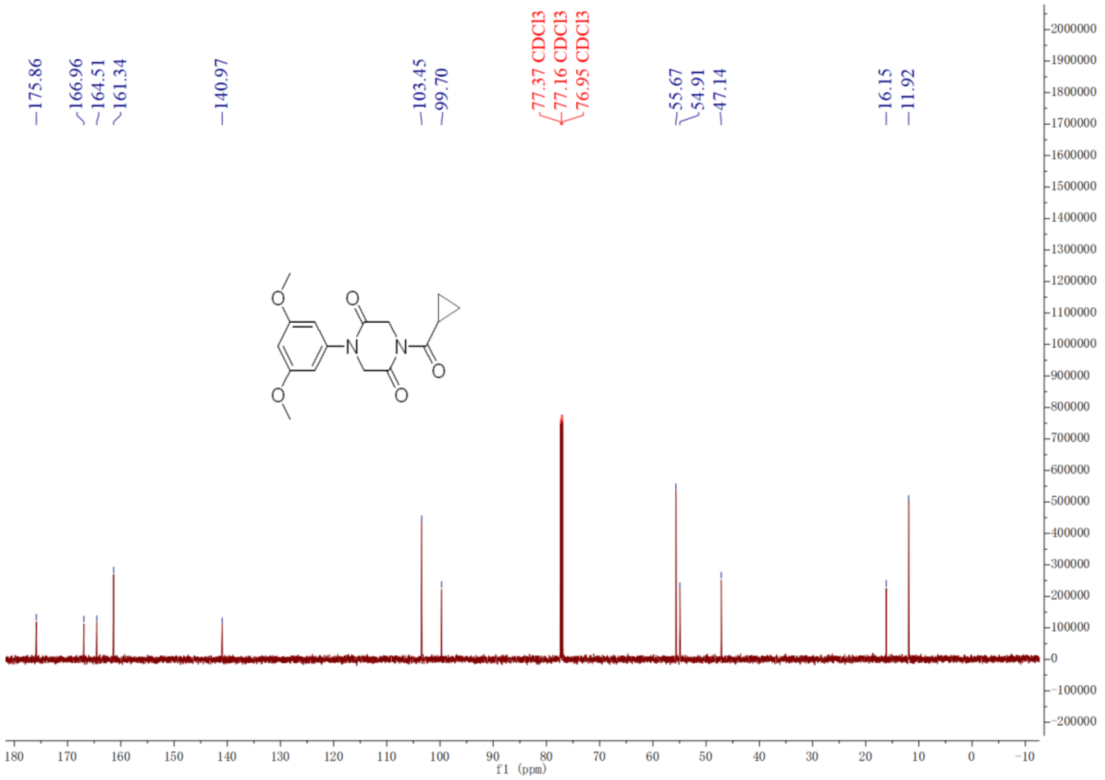

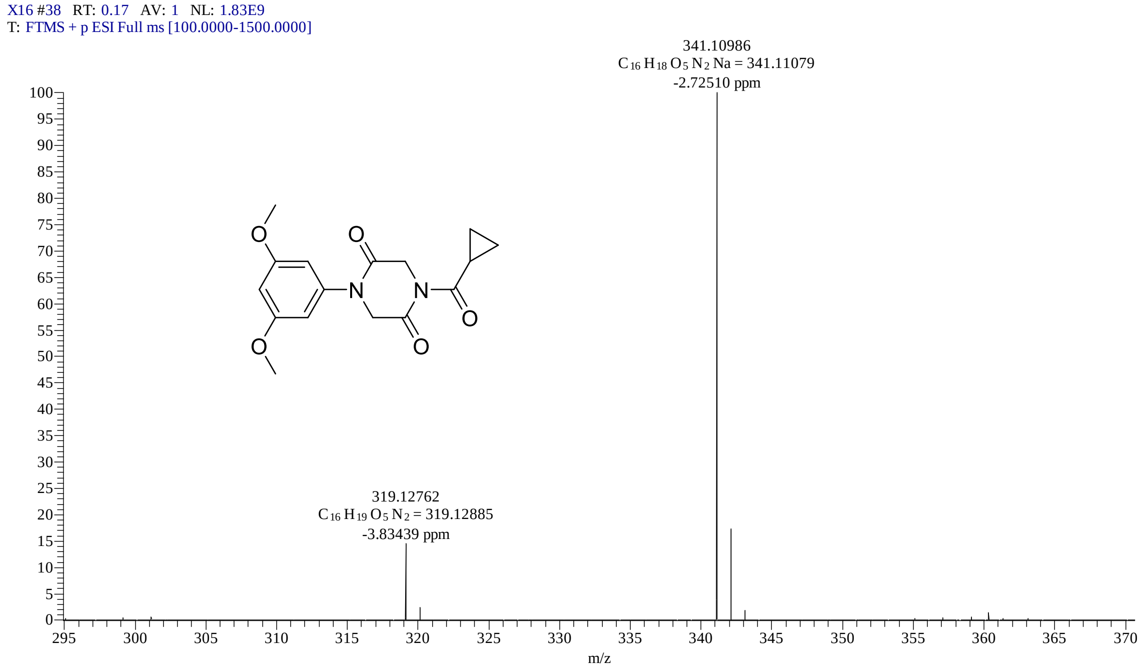

*1-(3,5-dimethoxyphenyl)-4-(3-cyclopentylpropanoyl)piperazine-2,5-dione (**9r**)*

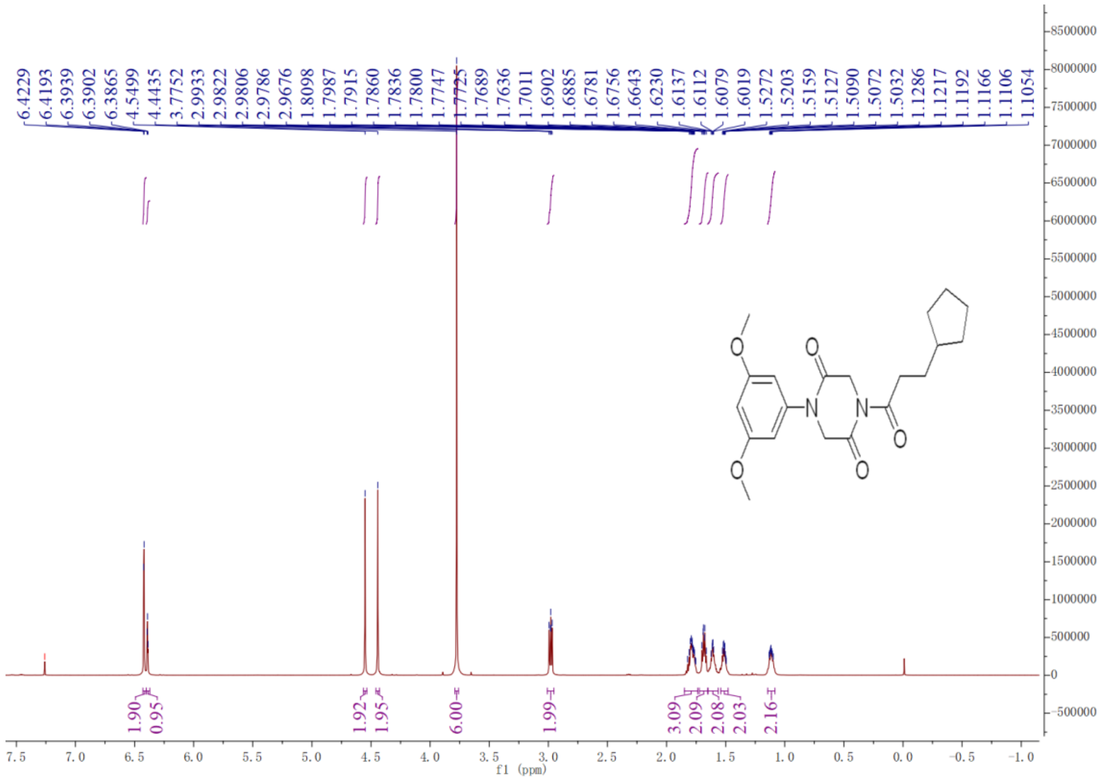
6.4229
6.4193
6.3939
6.3902
6.3865
4.5499
4.4435
3.7752
2.9933
2.9822
2.9806
2.9786
2.9676
1.8098
1.7987
1.7915
1.7860
1.7836
1.7800
1.7747
1.7725
1.7689
1.7636
1.7011
1.6902
1.6885
1.6781
1.6756
1.6643
1.6230
1.6137
1.6112
1.6079
1.6019
1.5272
1.5203
1.5159
1.5127
1.5090
1.5072
1.5032
1.1286
1.1217
1.1192
1.1166
1.1106
1.1054
1.90
0.95
1.92
1.95
6.00
1.99
3.09
2.09
2.08
2.03
2.16
f1 (ppm)

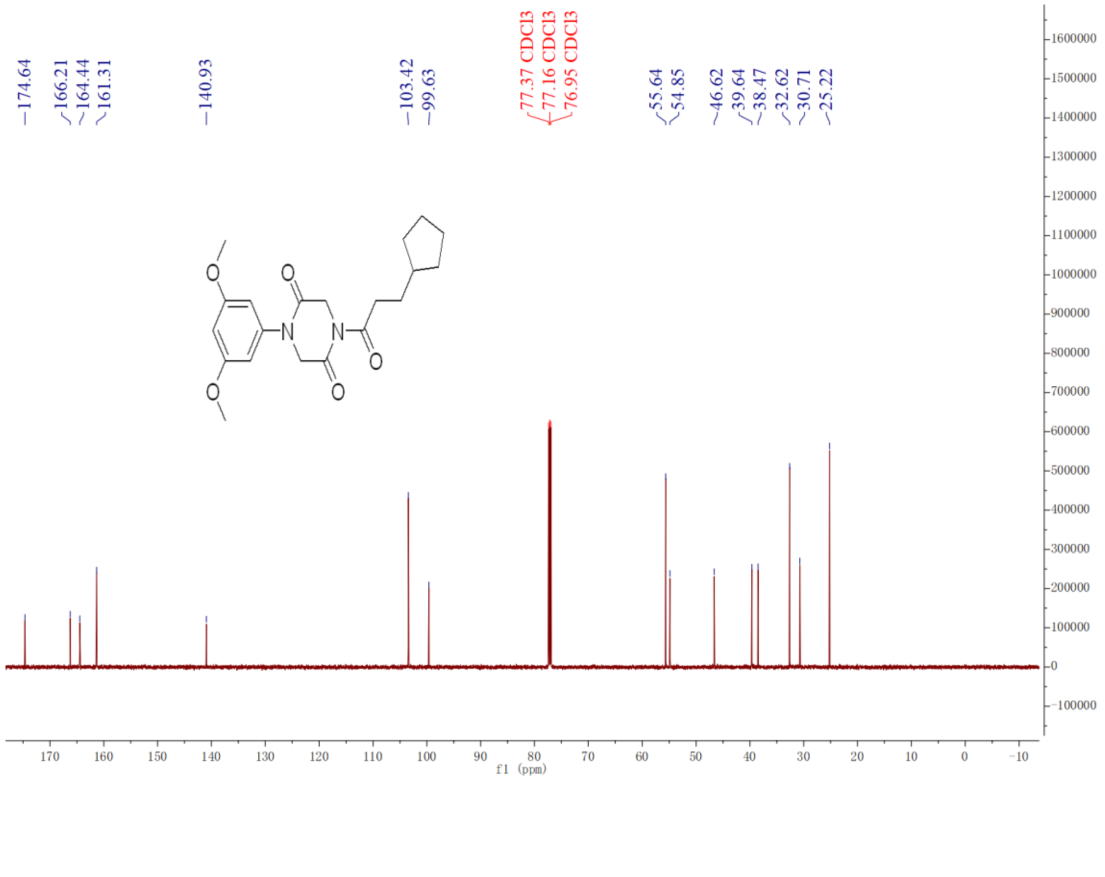
174.64
166.21
164.44
161.31
140.93
103.42
99.63
77.37 CDCl3
77.16 CDCl3
76.95 CDCl3
55.64
54.85
46.62
39.64
38.47
32.62
30.71
25.22
f1 (ppm)

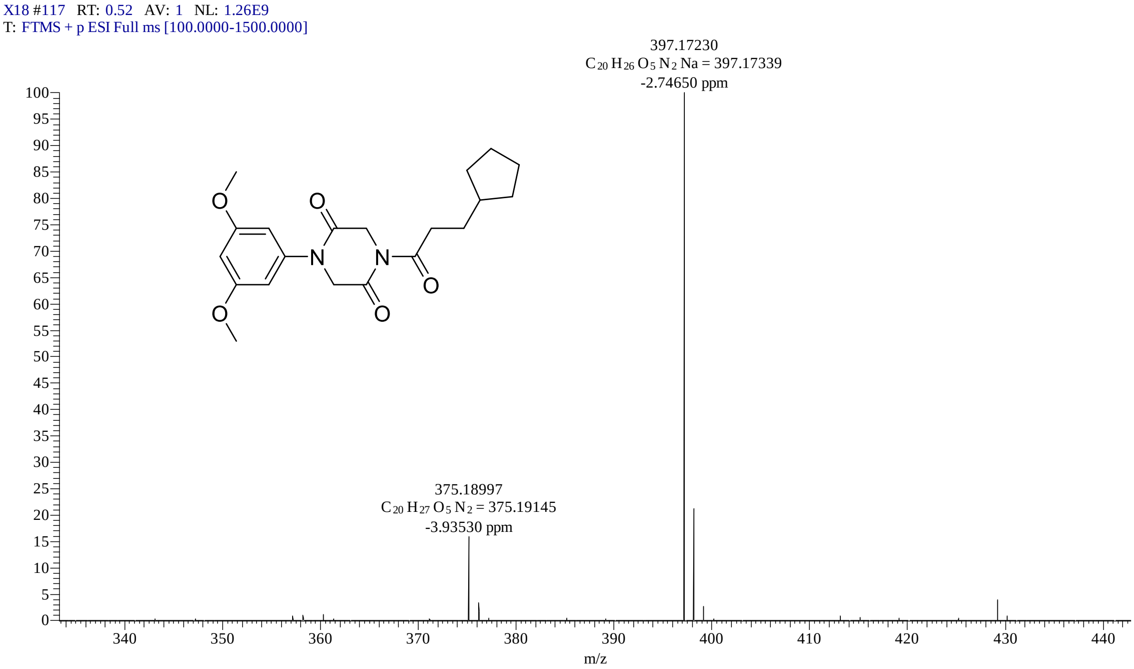

*1-(3,5-dimethoxyphenyl)-4-(2-methoxyacetyl)piperazine-2,5-dione (**9s**)*

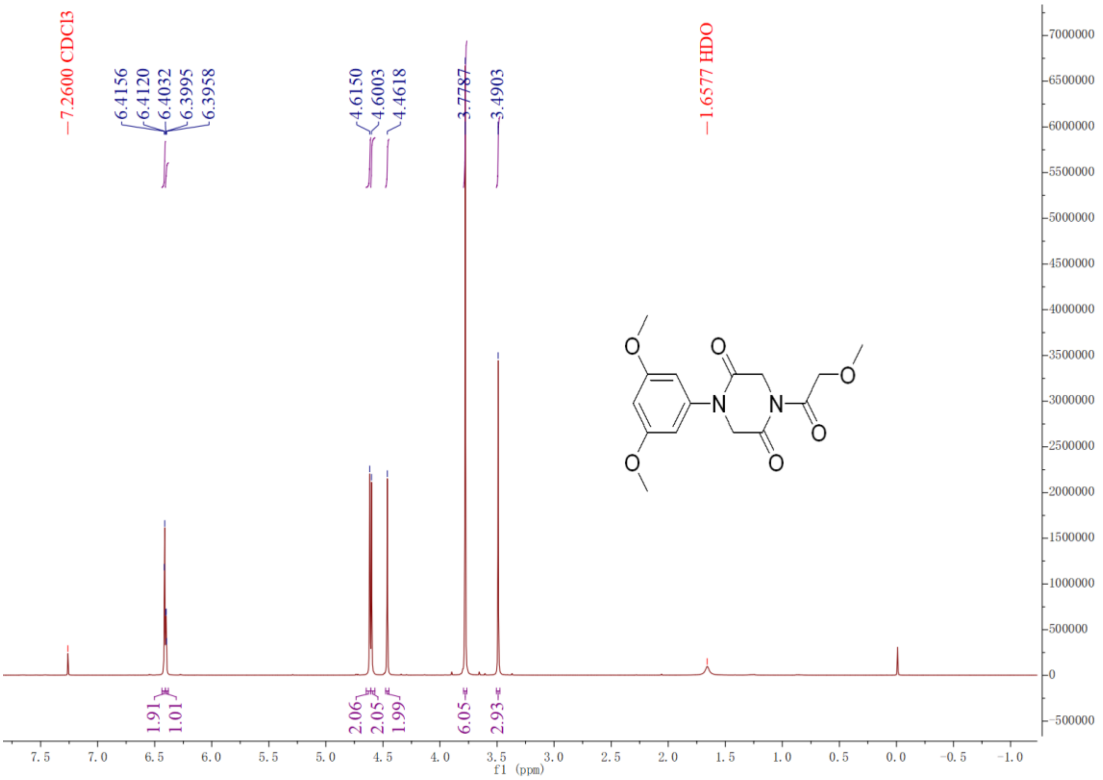

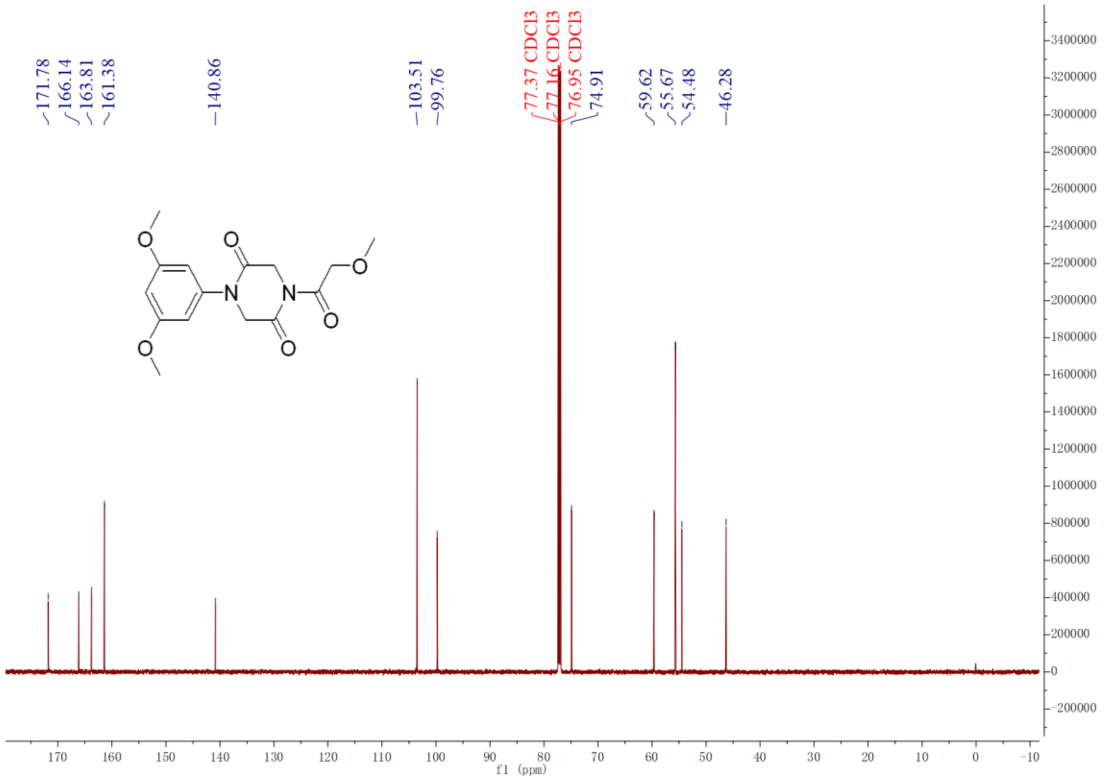

X20 #46 RT: 0.20 AV: 1 NL: 1.92E9
T: FTMS + p ESI Full ms [100.0000-1500.0000]

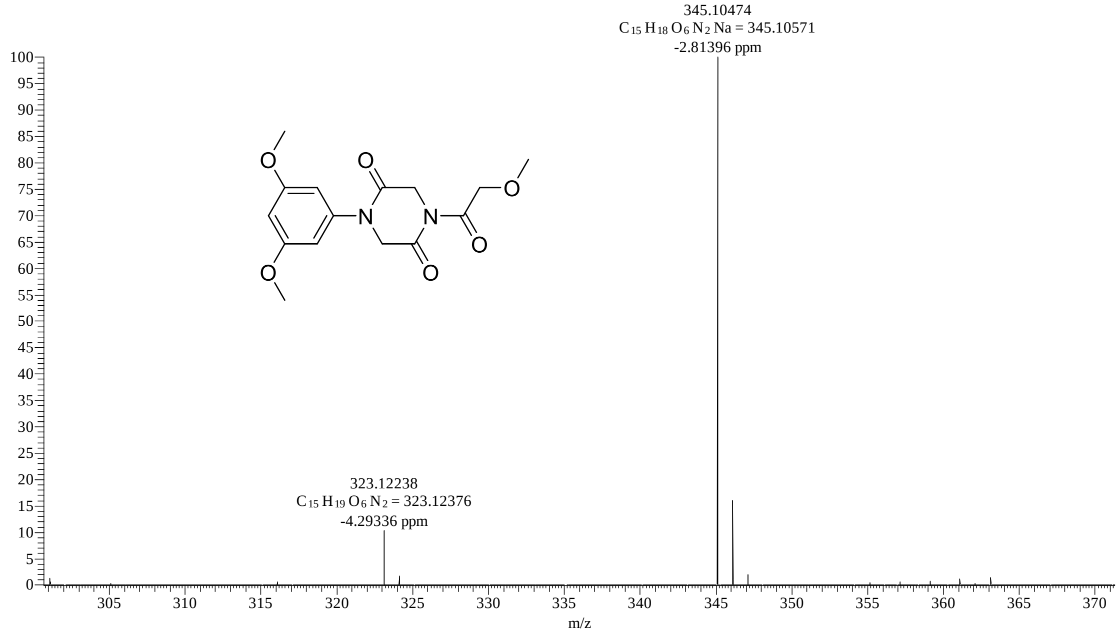

Supplement: Supplementary file 1 [file antioxidants-11-02014-s001.zip › antioxidants-1938264-supplementary.pdf]
